# Which oak provenances for the 22nd century in Western Europe? Dendroclimatology in common gardens

**DOI:** 10.1371/journal.pone.0234583

**Published:** 2020-06-10

**Authors:** Didier Bert, François Lebourgeois, Stéphane Ponton, Brigitte Musch, Alexis Ducousso

**Affiliations:** 1 INRAE, Univ. Bordeaux, BIOGECO, Cestas, France; 2 Université de Lorraine, AgroParisTech, INRAE, UMR Silva, Nancy, France; 3 CGAF-ONF, INRAE, Orléans, France; Universite du Quebec a Chicoutimi, CANADA

## Abstract

The current distribution area of the two sympatric oaks *Quercus petraea* and *Q*. *robur* covers most of temperate Western Europe. Depending on their geographic location, populations of these trees are exposed to different climate constraints, to which they are adapted. Comparing the performances of trees from contrasting populations provides the insight into their expected resilience to future climate change required for forest management. In this study, the descendants of 24 *Q*. *petraea* and two *Q*. *robur* provenances selected from sites throughout Europe were grown for 20 years in three common gardens with contrasting climates. The 2420 sampled trees allowed the assessments of the relationship between radial growth and climate. An analysis of 15-year chronologies of ring widths, with different combinations of climate variables, revealed different response patterns between provenances and between common gardens. As expected, provenances originating from sites with wet summers displayed the strongest responses to summer drought, particularly in the driest common garden. All provenances displayed positive significant relationships between the temperature of the previous winter and radial growth when grown in the common garden experiencing the mildest winter temperatures. Only eastern provenances from continental cold climates also clearly expressed this limitation of growth by cold winter temperatures in the other two common gardens. However, ecological distance, calculated on the basis of differences in climate between the site of origin and the common garden, was not clearly related to the radial growth responses of the provenances. This suggests that the gradient of genetic variability among the selected provenances was not strictly structured according to climate gradients. Based on these results, we provide guidelines for forest managers for the assisted migration of *Quercus petraea* and *Q*. *robur* provenances.

## Introduction

Genetic adaptation, phenotypic plasticity and migration are the three mechanisms by which populations face environmental variations, such as global warming [1–3]. Williams [4] defined the concept of “local adaptation” of populations as a population having a greater average fitness in its environment of origin than it would have in another environment. Environmental changes can, therefore, alter the hierarchy of population growth potentials. The time scale of climate change is a key issue, as adaptation and migration are very slow in trees, due to their longevity and sessile lifestyle. The persistence of trees under rapid climate change is therefore dependent principally on the phenotypic plasticity of individuals, and assisted migration in managed forests [5].

Two oak species (*Quercus robur* and *Q*. *petraea*) cover 35 000 km^2^, corresponding to 25% of forest area in France [6]. These oak forests are of considerable ecological, heritage and economic interest. The genetic resources of oaks have been intensively explored [7–9] and studied in provenance tests [10, 11], with a view to improving natural or artificial regeneration for forest management. Current genetic diversity in oak populations is derived mostly from post-glacial migrations: the Iberian refuge gave rise to the populations of Spain and Western France, whereas the populations currently found in eastern Europe spread from the Italian and Balkan refuges [8]. Furthermore, strong spatial structuring, at both regional and local scales, was revealed by studies based on isoenzymes and other genetic traits [9, 12]. However, lineage and haplotype do not significantly structure traits of interest in forestry, and provenances must therefore be tested [13]. These findings led to recommendations that local genetic resources should be used during natural regeneration, and that seeds from classified stands within the same region should be used in case of planting [14].

The practical implications of the genetic structure of oak stands for forest management under conditions of climate change remain unclear. Indeed, the adaptation of local oak forests to new, untested climatic conditions may require natural [3, 15], or assisted migration [16–18]. The effects of environmental changes can be studied by modelling [19, 20] or in field experiments involving provenance tests mimicking climate change [17, 21]. During post-glacial recolonization and the thousands of years that followed, natural oak populations gradually adapted to their local climate through selection acting on natural diversity. Phenotypic plasticity enables individuals to withstand rapid changes in climate in a certain range [3, 22]. Provenance tests are, therefore, highly suitable for (1) comparing the effects of climate on growth between provenances in a common garden, and (2) expressing the plasticity of the growth response through comparisons of common gardens in different climates. Ecological distance, also known as “ecodistance”, can be used to characterize the environmental change experienced by provenances between their geographic origin and the common garden [23]. In particular, the effect of both temperature and water supply differences between the original field site and the common garden can be assessed in provenance trials.

Many studies have been performed on *Quercus petraea* or *Q*. *robur* in European forests, to investigate the relationships between annual growth and climate [24–29]. These studies were based on large-scale observational networks of natural stands in which the provenance of the trees was not specifically controlled. Even in cases of strong regional climatic response patterns, it would not have been possible to separate the respective effects of local adaptation and genetic plasticity [27]. Studies addressing this question in provenance tests are still scarce, and most of those performed have focused on conifer species [30], with no such studies performed to date on European oaks. Several oak provenance trials have been established in European countries in recent decades [21, 31–33]. Many traits have been measured as proxies for fitness (review in [22]), but no dendroclimatic study has yet been performed on these relatively young oak trees. However, although tree-ring chronologies would be much shorter than those in classical dendroclimatic studies, assessments of the radial growth response of young trees experiencing year-to-year climate variations provide a unique opportunity to compare provenances and to infer their behavior in the longer term. However, obtaining tree-ring data is hard work and requires limited sampling within the framework of large provenance tests. The sampling scheme must be representative of the entire range of variation for the trait of interest. Finally, fewer provenances can be studied by such an approach, but their relationships to climate can be better characterized [34].

In this study, we aimed to evaluate the potential of assisted migration to facilitate the adaptation of oak forests in Europe. More specifically, the objectives of this study were (1) to analyze the tree growth-climate relationships of 24 *Q*. *petraea* and 2 *Q*. *robur* provenances selected throughout Europe and planted at three French sites (three common gardens) with contrasting climate conditions (wet-cold to dry-warm), (2) to assess the effect of ecodistance on the growth response to climate, (3) to provide guidelines for choosing suitable provenances adapted to future climate conditions. We hypothesized that 1) the response to climate highly differs between provenances and within common gardens and 2) the greater the ecodistance, the larger the likely response to climate.

## Material and methods

### Provenances in Europe

Seeds were collected from natural populations across the distribution range of *Quercus petraea* between 1986 and 1992 (an extensive description of the experiment is provided in [11]). In total, 116 populations of *Q*. *petraea* were selected, together with 17 populations of *Q*. *robur*. The acorns of each source population were harvested from the ground, over an area of 5 to 40 ha. Seed production is unevenly distributed and the acorns cannot be stored for more than one year. Acorns from the various provenances were therefore collected over four years (1986, 1987, 1989 and 1992) and sown every year of acorns collection, i.e. in four stages in the nursery.

**Fig 1:**
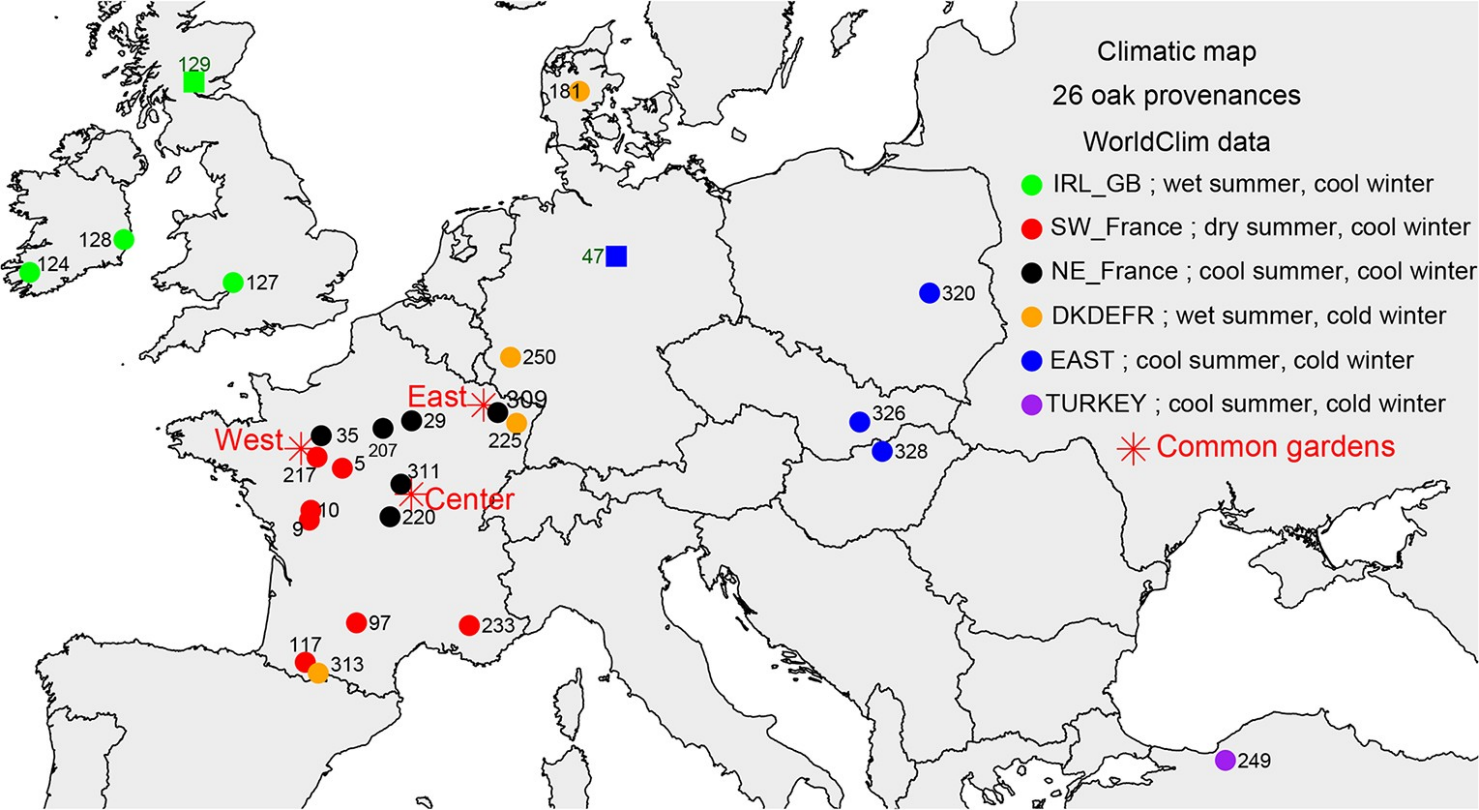
Location of common gardens (red stars) and source populations, color-coded according to climatic group (S2 Fig). Dots and black codes for *Quercus petraea*; squares and green codes for *Q*. *robur*. The map was generated using the R software. See text for details.

We used WorldClim [35] data to characterize the climatic conditions in the areas of origin of the provenances and to compare these conditions with those in the common gardens in which they were grown. WorldClim version 2 provides spatial average monthly climate data, with a resolution of about 1 km^2^, for minimum, mean, and maximum temperatures and for precipitation, for the 1970–2000 period. Potential evapotranspiration (PET) was calculated with the Turc method [36], and the monthly climatic water balance (WB) was defined as the difference between P and PET. This method provides an accurate estimate of fluctuations in water supply with a limited number of parameters and has been successfully used in many previous dendroclimatological studies [37, 38]. We reduced collinearity in the modelling process [39], by aggregating the climatic variables as follows: T1011 (mean temperature for months 10 and 11, i.e. previous October and November), T1202 (mean temperature for previous December to current February), T0304, T0506, T0708, T09, and water balances WB1011, WB1202, WB0304, WB0506, WB0708, WB09. Principal component analysis (PCA) and hierarchical ascending clustering according to Ward D2 method [40] were performed on these variables to compare the climates of the 26 source populations. PCA showed that source populations differed firstly in terms of their water balance during the growing season, and secondly in terms of mean temperature during the winter months (S1 and S2 Figs). Hierarchical ascending clustering identified six climatic groups (Fig 1, Table 1).

**Table 1:** Location and principal features of the source populations. Provenance code, name of provenance, number of trees sampled and provenance parameters.

Code	Provenance	Trees	Country	Latitude	Longitude	Elevation	Tyear (°C)	Pyear (mm)	Climatic group
127	Blakeney	129	Great Britain	**51.7831**	**-2.4981**	76	9.5	842	IRL_GB
129	Drummond Castle ***	130	Great Britain	**56.3431**	**-3.8489**	50	8.8	1037	IRL_GB
124	Killarney	90	Ireland	**52.0133**	**-9.5044**	50	10.0	1374	IRL_GB
128	Coolgreany	100	Ireland	**52.7607**	**-6.2607**	100	9.8	1002	IRL_GB
5	Blois 136	101	France	**47.5606**	**1.2606**	115	11.2	683	SW_France
9	St Sauvant	84	France	**46.3800**	**0.1244**	155	11.8	841	SW_France
10	Vouillé	73	France	**46.6042**	**0.1781**	130	11.7	735	SW_France
97	Grésigne	71	France	**44.0431**	**1.7489**	310	12.0	776	SW_France
117	Adé	97	France	**43.1467**	**-0.0158**	450	12.2	1067	SW_France
217	Bercé	90	France	**47.8131**	**0.3906**	165	11.1	716	SW_France
233	Vachères	72	France	**43.9833**	**5.6325**	650	11.6	822	SW_France
29	Traconne	89	France	**48.6386**	**3.6461**	200	10.0	649	NE_France
35	Bellême 67	96	France	**48.3989**	**0.5339**	220	10.5	721	NE_France
207	Fontainebleau 853	91	France	**48.4664**	**2.6608**	83	10.6	633	NE_France
220	Dreuille	84	France	**46.4567**	**2.9022**	290	10.8	721	NE_France
309	Bride	120	France	**48.8269**	**6.6103**	300	9.5	826	NE_France
311	Prémery	137	France	**47.1986**	**3.2775**	300	10.1	800	NE_France
181	Hørbylunde	84	Denmark	**56.1333**	**9.4217**	80	7.6	770	DKDEFR
225	Still	78	France	**48.5828**	**7.2619**	688	7.9	1059	DKDEFR
313	Bareilles	94	France	**42.8994**	**0.4311**	1300	8.7	1123	DKDEFR
250	Cochem	93	Germany	**50.0842**	**7.0519**	380	8.4	856	DKDEFR
47	Fallersleben ***	119	Germany	**52.3803**	**10.6933**	64	9.2	617	EAST
328	Nagybotany	61	Hungary	**47.9439**	**19.8511**	400	8.4	573	EAST
320	Kozienice	92	Poland	**51.5456**	**21.4828**	150	8.1	521	EAST
326	Obora	78	Slovakia	**48.6106**	**19.0767**	350	8.0	707	EAST
249	Bolu (Ayikayasi)	67	Turkey	**40.9167**	**31.6667**	1200	7.2	654	TURKEY

Tyear is the annual mean temperature, and Pyear is the annual total precipitation. Climatic groups were defined with WorldClim data (see text and S2 Fig). The distribution of tree numbers by common garden and provenance is given in S1 Table.

*** for *Quercus robur*.

**Fig 2:**
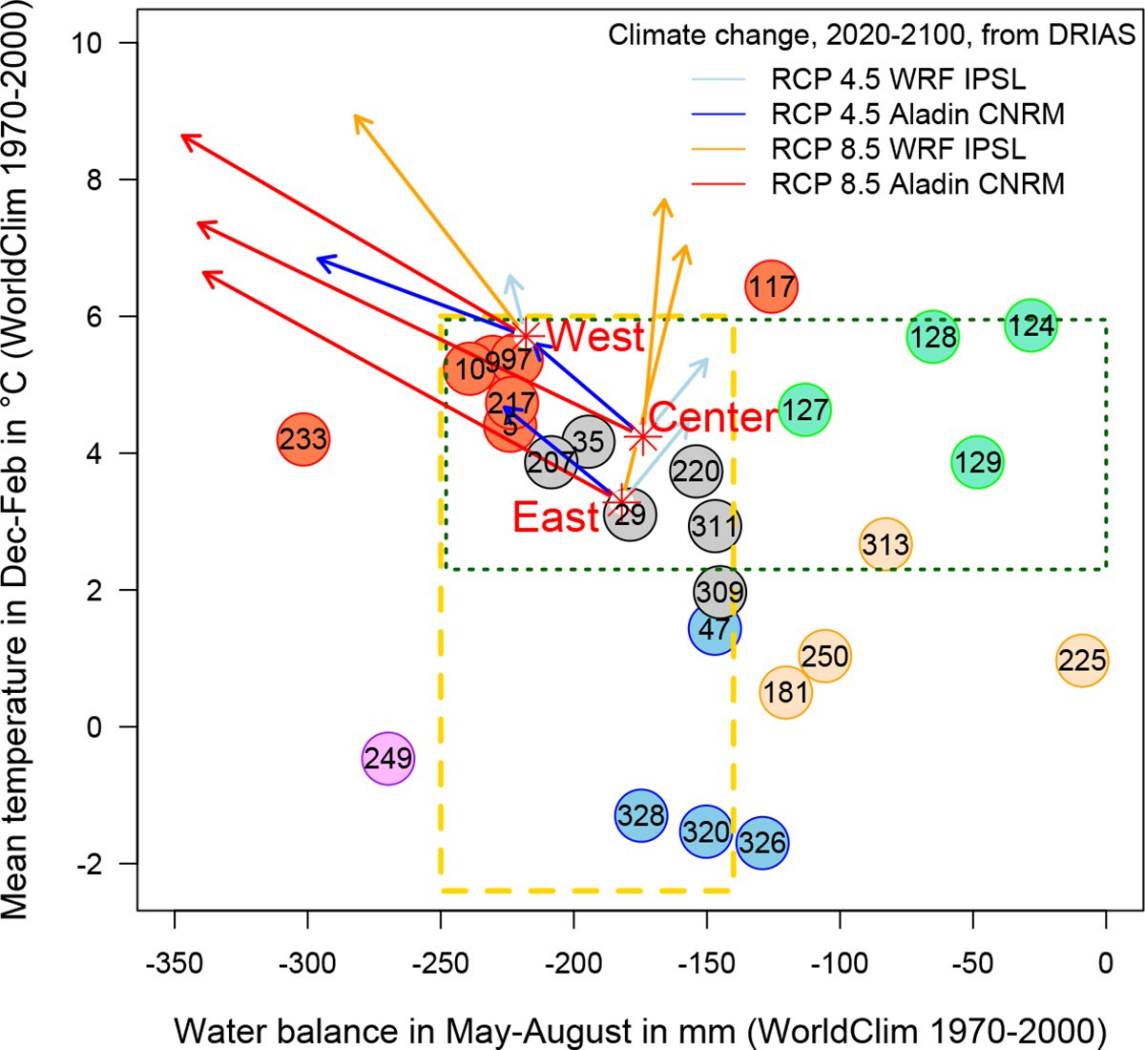
Mean temperature in winter (December to February) and climatic water balance during the growing season (May to August) at the site of the source population. The color of the circles surrounding the provenance code indicates the climatic group (S2 Fig). The three common gardens are indicated with orange dots and named “West”, “Center”, and “East”. The rectangle outlined with a dashed yellow line shows the warming gradient when a provenance was moved to the common gardens, and the rectangle outlined with a green dotted line indicates climatic drying. Provenances 250 and 181, for example, were both moved to common gardens that were warmer and drier (both gradients) than their sites of origin. The arrows indicate the future climate conditions predicted for each common garden in 2100 under two scenarios of greenhouse gas emissions (RCP) and based on two different climatic models for France (WRF and ALADIN-Climat). See the text for details.

In their areas of origin, some provenances grow with almost no water limitation during the summer (Fig 2; *e*.*g*. 225 from eastern France France, 124 from Ireland, 129 from Scotland). By contrast, other provenances are subject to a low water supply (*e*.*g*. 233 from southern France, 249 from Turkey). The provenances subjected to the lowest winter temperatures were the most eastern ones (*e*.*g*. 249 from Turkey, 320 from Poland, 326 from Slovakia, 328 from Hungary). The values obtained covered gradients of 300 mm for summer water balance and 8°C for winter temperature.

### Common gardens in France

After three growing seasons in nursery, the seedlings were planted out in common gardens at three experimental sites in France along a west-east gradient (Fig 1). These common gardens are called “Petite Charnie” (referred to as “West”, lat. 48.087; long. -0.159; alt. 150 m), “Vincence” (“Center”, lat. 46.969; long. 3.633; alt. 235 m), and “Sillegny” (“East”, lat. 48.997; long. 6.124; alt. 201 m). At each experimental site, a complete randomized block design was used to account for within-site variation within each stage, and incomplete micro-blocks were nested in the blocks. Each micro-block consisted of eight plots of 24 oaks planted 1.75 m x 3 m apart. The access of researchers from INRAE (Institut National de Recherche pour l’Agriculture, l’alimentation et l’Environnement) to field site managed by ONF (Office National des Forêts) was approved by “Research Convention 2013–2016 ONF-INRAE Genetic Variability of Fagaceae”.

**Fig 3:**
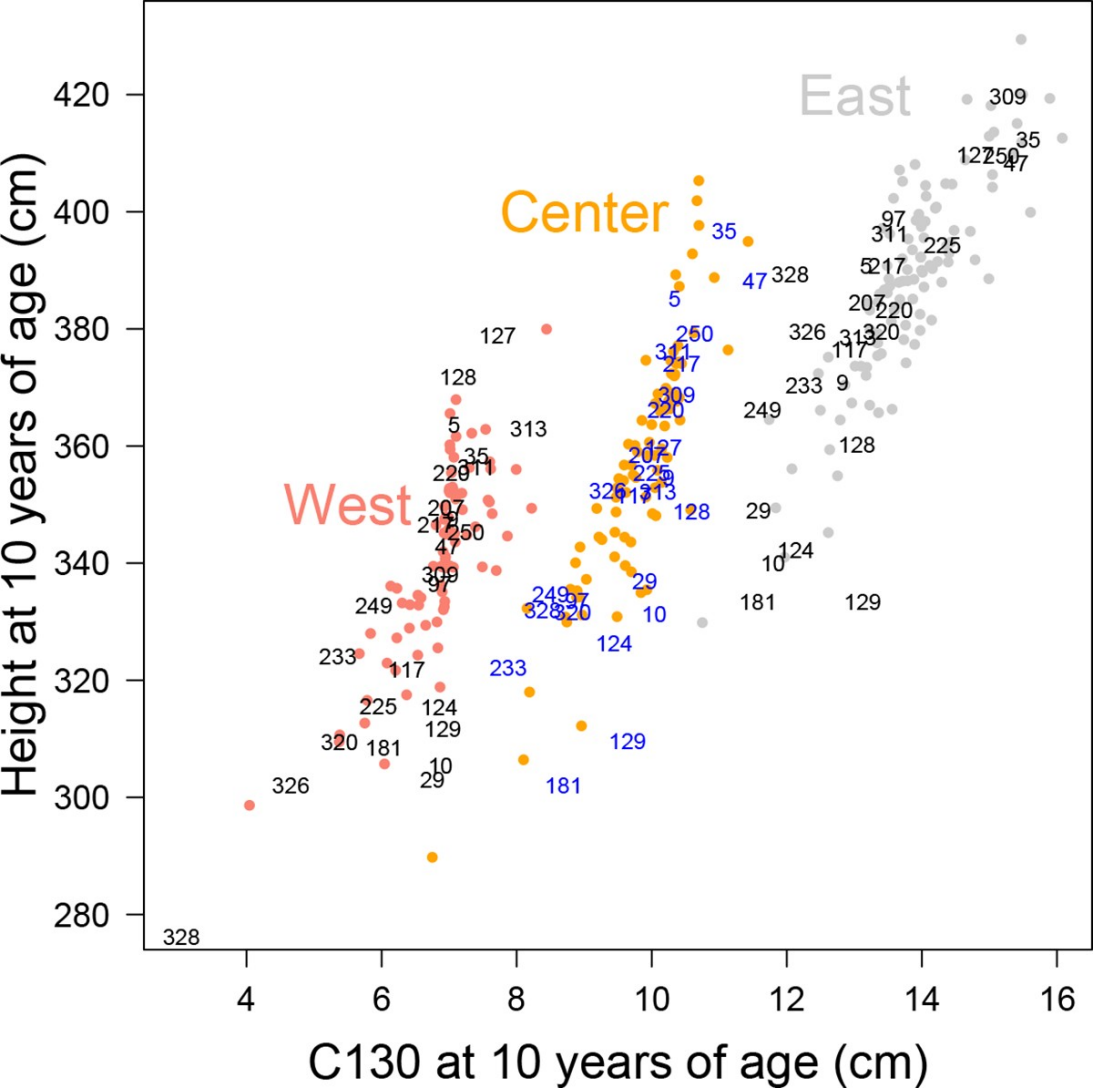
Adjusted mean dimensions of provenances at the age of 10 years, from complete inventory for each of the three common gardens, taking into account the effects of stage. The numbers give the codes of the provenances included in this study, and the colored dots correspond to other provenances present in the common gardens. The codes for Center are shown in blue to facilitate their identification on the figure.

For this study, we selected 24 provenances of *Q*. *petraea* and two of *Q*. *robur* covering the European climate range (annual mean temperature of about 7–12°C and total precipitation of about 500–1400 mm; Table 1) and the growth variability [11]. These provenances were selected on the basis of height and circumference at breast height (C130) at the age of 10 years, as recorded in complete inventories (Fig 3). Later on, the data for another complete inventory was available for 20-year-old trees. We checked that the ranking of provenances was largely conserved between these two time points. For example, for stage planted in 1989 at West with 57 provenances: the correlation between the results for 10-year-old and 20-year-old trees was r = 0.87 for total height, and r = 0.87 (*p*<0.0001) for circumference at breast height, at provenance level.

**Fig 4:**
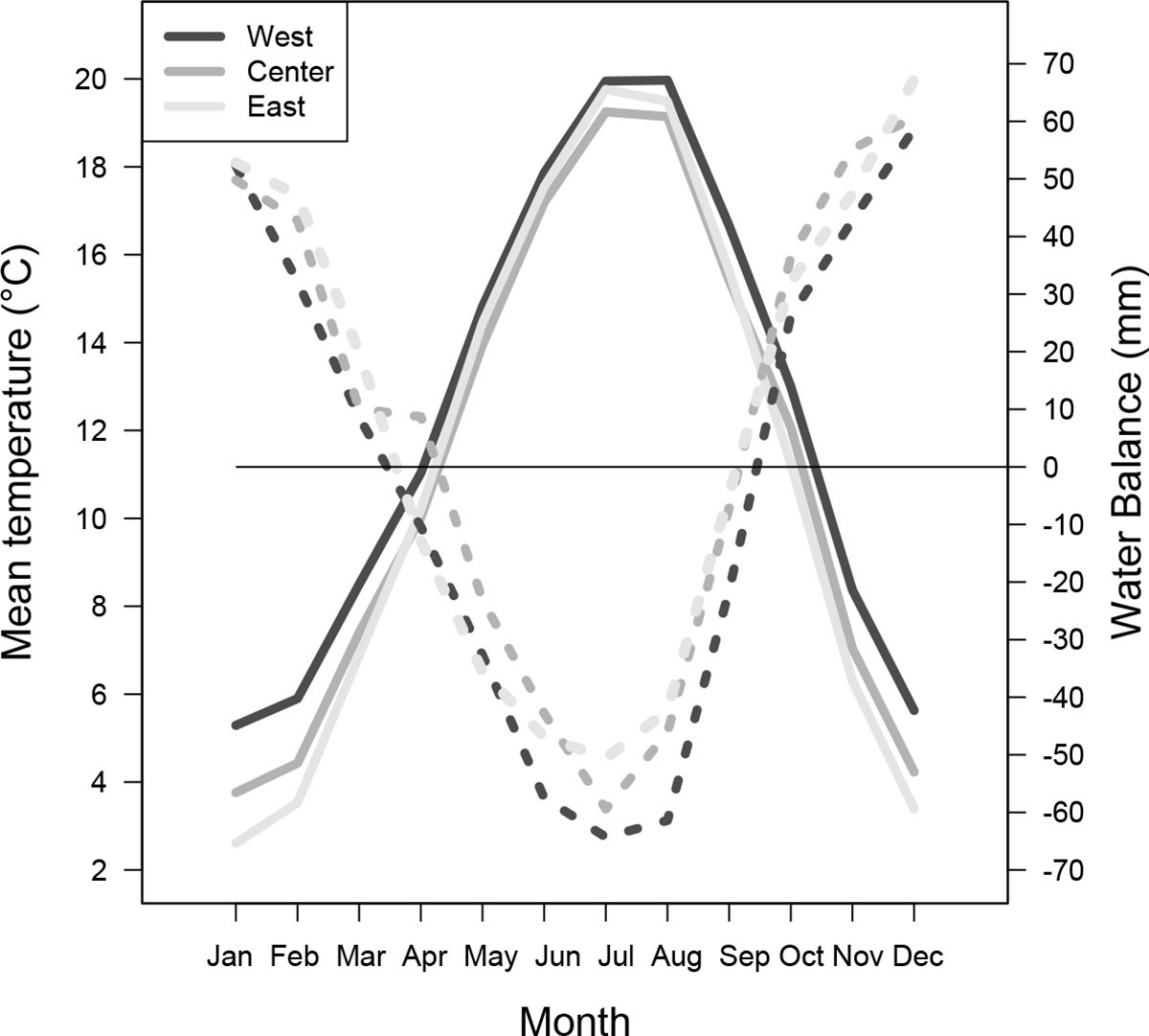
Mean monthly temperature (°C) and climatic water balance (mm) calculated for the 1987–2016 period with climate data from weather stations close to the common gardens. Solid lines are used for temperature and dashed lines for water balance. Mean annual temperature (T) and annual total precipitation (P) at West: T = 12.2°C, P = 685 mm, Center: T = 11.2°C, P = 789 mm, and East: T = 10.9°C, P = 730 mm.

To characterize accurately the local climate conditions, climate data were obtained from the “Météo-France” weather stations closest to each common garden: Le Mans (lat. 47.946; long. 0.184; alt. 48 m; 30 km from West), Marzy (lat. 46.997; long. 3.113; alt. 220 m; 40 km from Center) and Augny (lat. 49.076; long. 6.128; alt. 174 m; 9 km from East). Monthly aggregates of mean daily temperature (T) and precipitation (P) were obtained for the period from 1987 to 2016. Mean annual temperature (Ty) and total precipitation (Py) were 12.3°C and 685 mm, respectively, for West, 11.2°C and 788 mm, respectively, for Center, 11.0°C and 725 mm, respectively for East. As with the provenances climate data (see above), climate data from the three common gardens were used to calculate PET, WB and then aggregated by two or three months. The three common gardens differed in winter temperature and growing season water balance (Fig 4). Winter temperatures decreased by 2.5°C and summer dryness decreased by 30% from West to East. The common garden farthest west is located in the warmest and driest zone of the natural range of the studied oak species in France [41] as these species are not represented in Mediterranean climates (South of France).

The soils developed on shale rock at West, and marls at Center and East all are rather acidic (pH 4.5 to 5.5). The soil at West has a sandy loamy texture and that at Center and East is a silty clay. The soils at West and East are well drained, whereas Center presents a locally impermeable deeper layer with a higher clay fraction, leading to water stagnation in fall and winter (periodically waterlogged soil). The deep rooting observed in such soils [42, 43] and the texture of these soils result in a water-holding capacity of up to 130–140 mm [44]. These soils provide favorable conditions for oak growth [45].

### Ecodistance and future climatic conditions

The 26 provenances were experimentally migrated from their original conditions to the common gardens, resulting in a shift in climate. The provenances thus experienced a double ecodistance gradient: warming/cooling in winter and increasing/decreasing drought in summer (Fig 2). For example, provenance 225 (from eastern France) was grown in conditions with 200 mm less rainfall in summer and winter temperatures that were 5°C higher at West. For the eastern provenances (320 from Poland, 326 from Slovakia, 328 from Hungary), the decrease in summer water supply was smaller (only about 80 mm) but the increase in winter temperatures was larger (+7°C). Conversely, summer water supply was about 80 mm more favorable for provenances 233 (southern France) and 249 (Turkey), but 249 experienced a 6°C increase in winter temperatures, whereas winter temperatures in the area of origin and the common garden were similar for provenance 233.

The calculated ecodistances were compared with the climate change predicted by climatic models. Two of the four scenarios proposed by IPCC [46] were considered in this study: RCP4.5 (stabilization of emissions after 2050, warming by 1.0 to 2.6°C in 2100) and RCP8.5 (high emissions, warming by 2.6 to 4.8°C). Regional climate models (RCMs) can predict different values for climatic parameters. We therefore also considered two RCMs: (1) the Weather Research & Forecasting Model (WRF) run by the “Institut Pierre-Simon Laplace”, and (2) the ALADIN model run by the “Centre National de Recherches Météorologiques”. Both provide climatic data for an 8 km geographic grid in France via a web service: DriasLes futurs du climat (http://www.drias-climat.fr). The daily temperature and precipitation predictions of the two RCMs for the three common gardens were downloaded, and PET and water balance were calculated as described above (Fig 2). By 2100, the region of West may be 3°C warmer in winter, and 130 mm drier in summer (according to the ALADIN model and RCP8.5), corresponding to conditions similar to those currently experienced along the French Mediterranean coast. The ALADIN model generally predicted larger changes than the WRF, particularly under the RCP8.5 scenario. However, the ecodistance gradients described above are greater than the predicted climate changes: e.g., eastern provenances (320, 326, 328) experienced a 7°C increase in winter temperatures when grown at West, whereas climate change prediction models suggested that West may become +3°C warmer over the next 80 years. Thus, the ecodistances were large enough to study the likely effects of strong climatic changes. However, there are currently no *Quercus petraea* provenances growing in conditions corresponding to the future climate predicted by RCP8.5.

### Tree sampling and growth data

During the first thinning in the provenance tests, one in two trees were cut down, providing an opportunity to collect stem discs from a height 0.3 m above soil level, between 2010 and 2017 (trees aged approximately 25 years). Stem discs were sanded (grid 80), and a wooden bar of 42 mm large x 24 mm thick was cut through the diameter of each stem disc. From each bar, a 2 mm-thick lath was finally shaped with a double-bladed saw. The laths were exposed to X rays to obtain photographic negatives [47], which were scanned. The ring width (RW) and wood density were measured by manually marking the tree-ring limits with WinDENDRO software on the images of the negatives [48]. For this study, we required only RW data, which were obtained from the two radii of each bar. The RW series were visually cross-dated with specific pointer years, i.e. narrow rings in 1995, 1998, 2003 and wide rings in 2004 and 2007 [27, 41]. RW values were then converted into basal area increment (BAI) values for growth assessment, and the two radii of the bar were averaged. For trees with two or three stems close to ground level, BAI series for the discs of the various stems were summed by year.

The relationships between climate and growth are weakened by social competition within oak stands [29, 38, 49]. We ensured that only the most climate-sensitive individuals were retained for analysis, by removing the suppressed trees with a method applied to each of the 78 combinations (26 provenances x 3 common gardens). This method assumes that tree size results from many different effects, making it possible to model the distribution with a log-normal function [50]. For a given provenance in a given common garden, the function gives BA_M_, corresponding to the abscissa of the maximum of the fitted frequency (i.e the size of the stem disc found in the largest number of trees):

$$BA_{M}=exp(\mu –\sigma^{2})\;where\;\mu \;is\;the\;mean\;of\;BA\;and\;\sigma^{2}\;the\;variance\;of\;BA$$

All the trees with a BA lower than BA_M_ were removed from the dataset (29% of total trees). Finally, with the 2420 dominant or codominant trees, we were able to analyse 33 954 tree rings (S1 Table). Each individual series of BAI values was then fitted with a cubic spline using the DENDRO function which is based on dplR package in R [51]. The rigidity of the cubic splines on the circa 20 years time-series was fixed to 7 years (one third of the series length) and the wavelength cutoff was 50%. Then, the crude values of BAI were divided by the value of the cubic spline for the calculation of 78 detrended master chronologies of Growth Index (GI). These 78 master chronologies were used to determine climate-tree growth relationships for the common period 1996–2010.

### Statistical analyses

Bootstrapped correlation coefficients (BCCs) were calculated with each master chronology as a dependent variable and 12 climatic regressors: 12 aggregated temperatures and water balance values (as described in the Provenances in Europe section) [52, 53]. Some climatic series of source populations or common gardens included slight positive or negative trends over time, which can lead to spurious correlations with the growth time-series [54]. We therefore adjusted all climate series (1987–2016, i.e. the whole period of growth of studied oaks) with a cubic polynomial and detrended them taking the difference between climatic values and the polynomial. Basically, a response function of climate over a 15-year period (1996–2010) is a Pearson correlation between annual growth (GI) and a climate variable (S3 Fig). In order to consolidate the correlation estimate and test its significance, it was calculated 10,000 times by bootstrap method and the correlation was then called BCC. The data analysis table includes 26 provenances x 3 common gardens = 78 lines and 12 climatic variables, i.e. 936 BCCs. PCA analyses were then performed on the BCCs to highlight the structure of the response to climate [40]. Principal components were calculated from the variance-covariance matrix because the descriptors were of the same kind and of the same order of magnitude.

## Results for growth-climate correlations

The master chronologies were first characterized with two usual dendrochronological parameters (S2 Table). The mean inter-series correlation (rbar) quantifies the strength of the signal common to all trees [55]. Most of the 78 master chronologies showed values between 0.3 and 0.8, i.e. high correlation between trees of a given provenance in a given common garden. The Expressed Population Signal (EPS) varies within 0 to 1 and quantifies the degree to which the chronology expressed the population chronology [56]. An overwhelming majority of the master chronologies had EPS values between 0.85 and 0.99 which indicated how closely the number of sampled trees in the present study allowed to represent a hypothetical chronology based on an infinite number of trees. For a given tree population, EPS value depends on the number of sampled trees and on the species. For shade-intolerant species like *Q*. *petraea*, the minimum threshold of 0.85 can be reached with about 10 trees [57] and the studied provenances were represented by 30 trees on an average (S1 Table). Eventually, the quality of master chronologies was a guarantee of the robustness of the analyses that followed.

### Site effect

**Fig 5:**
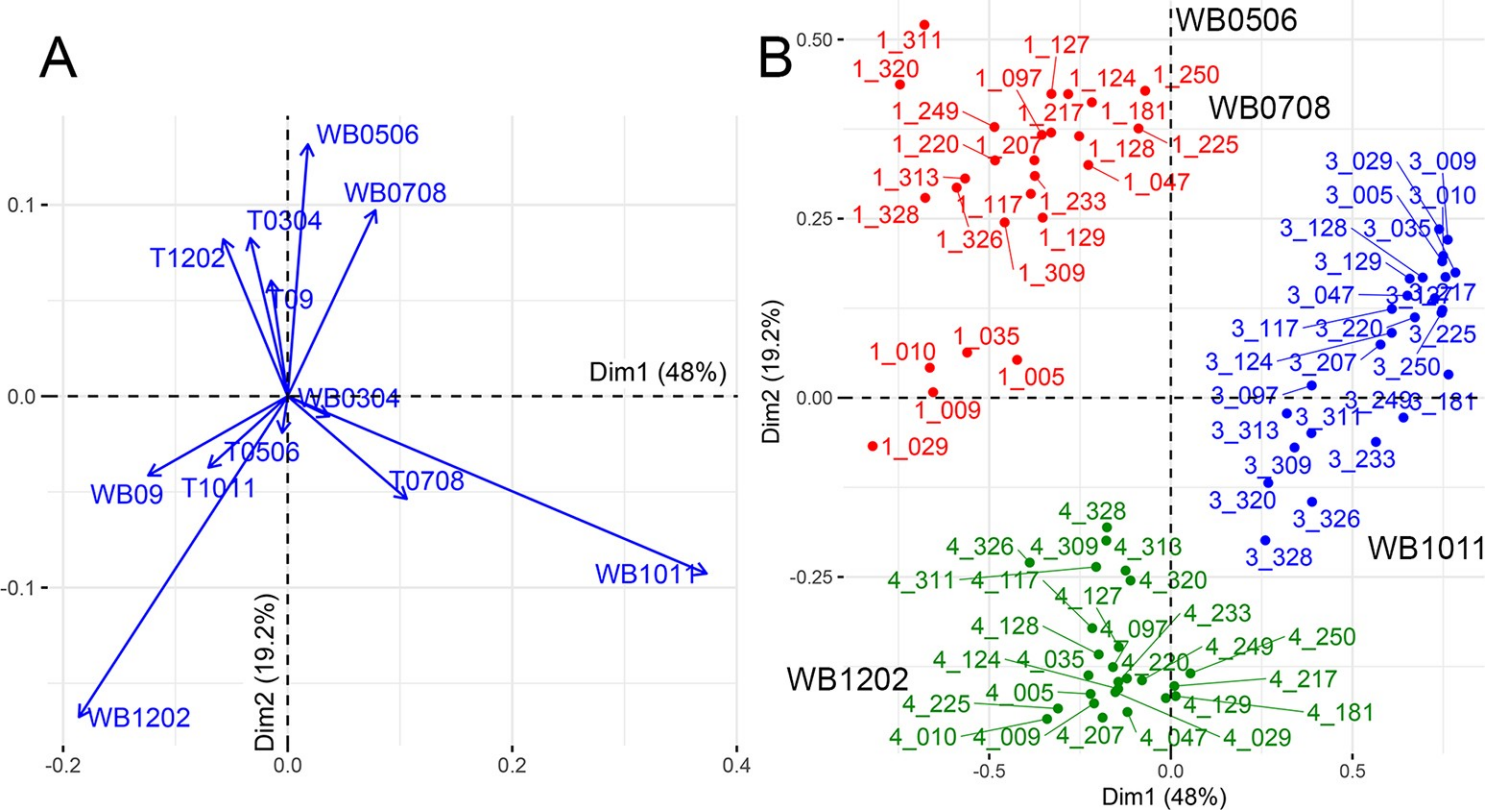
A. Variable scatter plot of the principal component analysis (PCA) performed on bootstrapped correlation coefficients (BCCs) of the 26 provenances with principal components 1 (*x*-axis) and 2 (*y*-axis). The climatic variables are T (temperature) and WB (water balance) in July and August (e.g., T0708), September (T09), or October and November of the preceding fall (e.g., WB1011). The most significant variables are those closest to the correlation circle. B. Provenance scatter plot of the same PCA with principal components 1 (*x*-axis) and 2 (*y*-axis). The four most significant variables are also displayed: WB1011 (water balance in the previous Oct-Nov), WB1202 (water balance in the previous Dec and current Jan-Feb), WB0506 (water balance in current May-June), WB0708 (water balance in July-Aug). The red points are for West, blue points are for Center, and green points are for East. Codes: 3_328, for example, in which the initial 3 indicates the common garden (1 for West, 3 for Center, 4 for East) and 328 is the code for the provenance.

In the PCA on BCCs, axes 1 and 2 (PC1 and PC2) accounted for 48% and 19.2% of the total variance, respectively (Fig 5A). Overall, temperature played a lesser role than water balance in the climatic responses of oak provenances. The main structuring climatic factors explaining the distribution of BCCs on PC1 were previous fall (WB1011) and winter (WB1202) water balance (correlation with PC1 r = 0.96 and r = -0.68, respectively). PCA also clearly identified three subsets exactly matching the three common gardens (Fig 5B). Ordination along the first axis strongly discriminated common garden Center with positives co-ordinates, and the key factor was linked to WB1011. The other two common gardens (West and East) had negative positions and the discriminating factor was WB1202. PC2 revealed a clear separation between West-Center and East, characterized by positive co-ordinates for West-Center and negative co-ordinates for East. The main climatic factors associated with PC2 were spring (WB0506) and summer (WB0708) water balances and winter temperature (T1202) (correlation with PC2 r = 0.77 and r = 0.61 for WB, and r = 0.56 for T). Thus, West and Center responded more strongly to water supply during the growing season (*i*.*e*. higher BCC) than East. At Center, water balance in the fall (WB1011, WB09) also played an important role, probably due to the temporarily waterlogged soil.

**Fig 6:**
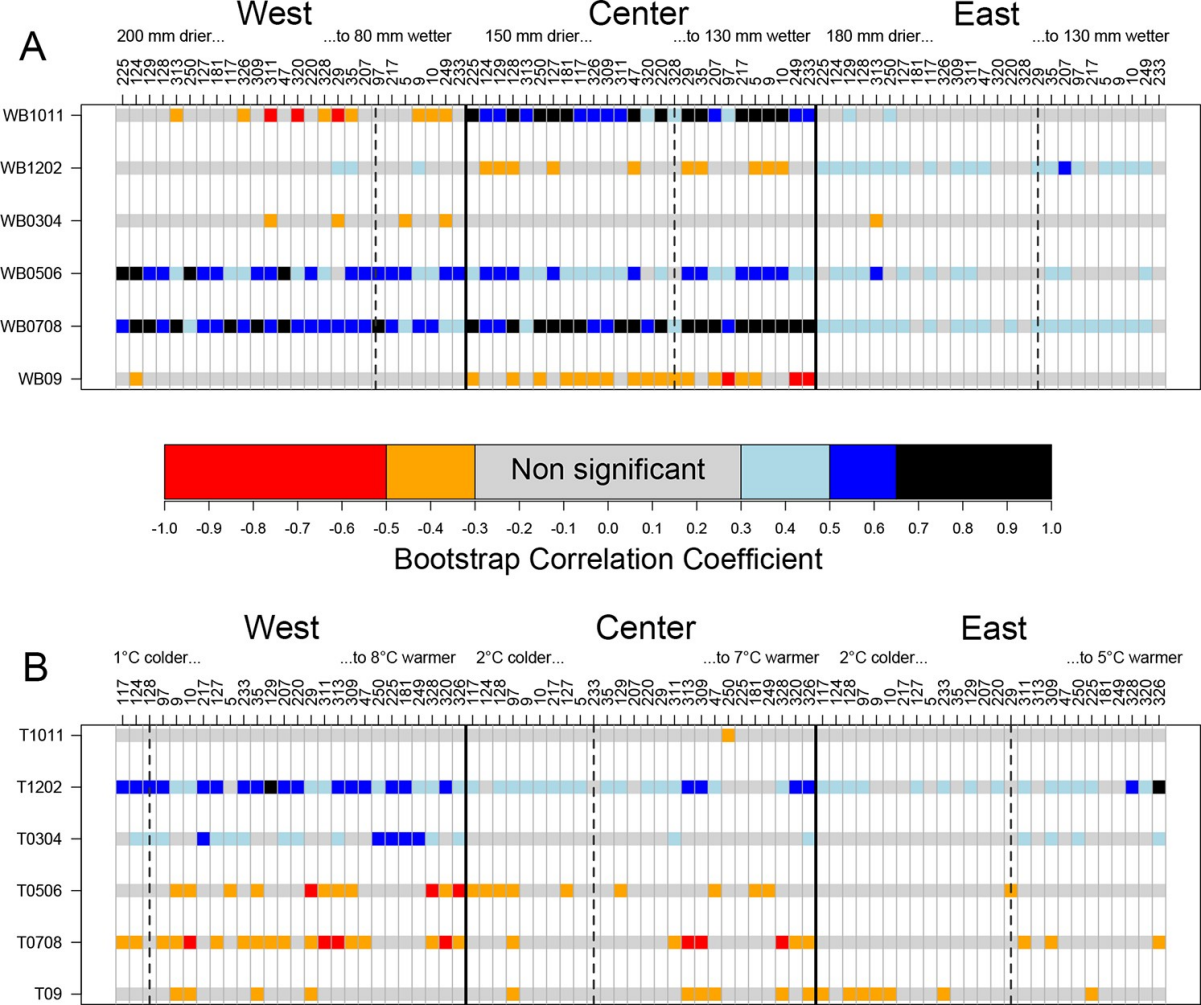
Bootstrapped correlation coefficients (BCCs), by common garden and provenance (columns) and climatic variables (rows): water balance, in mm (A) and temperature, in °C (B). Color codes indicate the BCCs, categorized as indicated by the central colored bar. A high BCC indicates high sensitivity to the climatic variable. Codes for climatic variables are T (temperature) and WB (water balance), with numbers indicating the months (e.g. 0708 means July to August). For each common garden, provenances were sorted by ecodistance, calculated with the water balance in May to August in figure A (mm), and temperature in December to February in B (°C). The order of the provenances is thus given by their projection onto each of the axes of Fig 2. The dashed vertical lines indicate an ecodistance of 0.

Sensitivity to drought during the growing season was generally significantly positively correlated with vigor expressed in terms of radial growth (i.e., C130, at West and Center; Table 2, S4 Fig). The most vigorous provenances were generally more likely to display lower growth rates during water stress at the driest common garden and at the waterlogged one. The drought sensitivity of provenances at the easternmost common garden (East) was low (Fig 6A) and not significantly correlated with vigor, which was also not correlated with mean winter temperature (T1202), resulting in fewer restrictions on the choice of provenances.

**Table 2:** Pearson’s correlation coefficient (r) and associated probability (p) between bootstrapped correlation coefficients (BCC) for WB0508 and T1202 and vigor expressed as circumference at breast height and total height at the age of 10 years (C130 and Ht10, respectively) for the three common gardens.

	West		Center		East	
WB0508	r	*p*	r	*p*	r	*p*
C130	0.483	0.012 *	0.453	0.020 *	0.279	0.166
Ht10	0.312	0.120	0.247	0.224	0.072	0.727
T1202						
C130	0.089	0.666	0.056	0.784	0.106	0.605
Ht10	0.068	0.742	0.137	0.505	0.264	0.191

*N* = 26 provenances (see text for details and S4 Fig).

*: significant at the 0.05 level.

### Provenance effect and ecodistance

**Fig 7:**
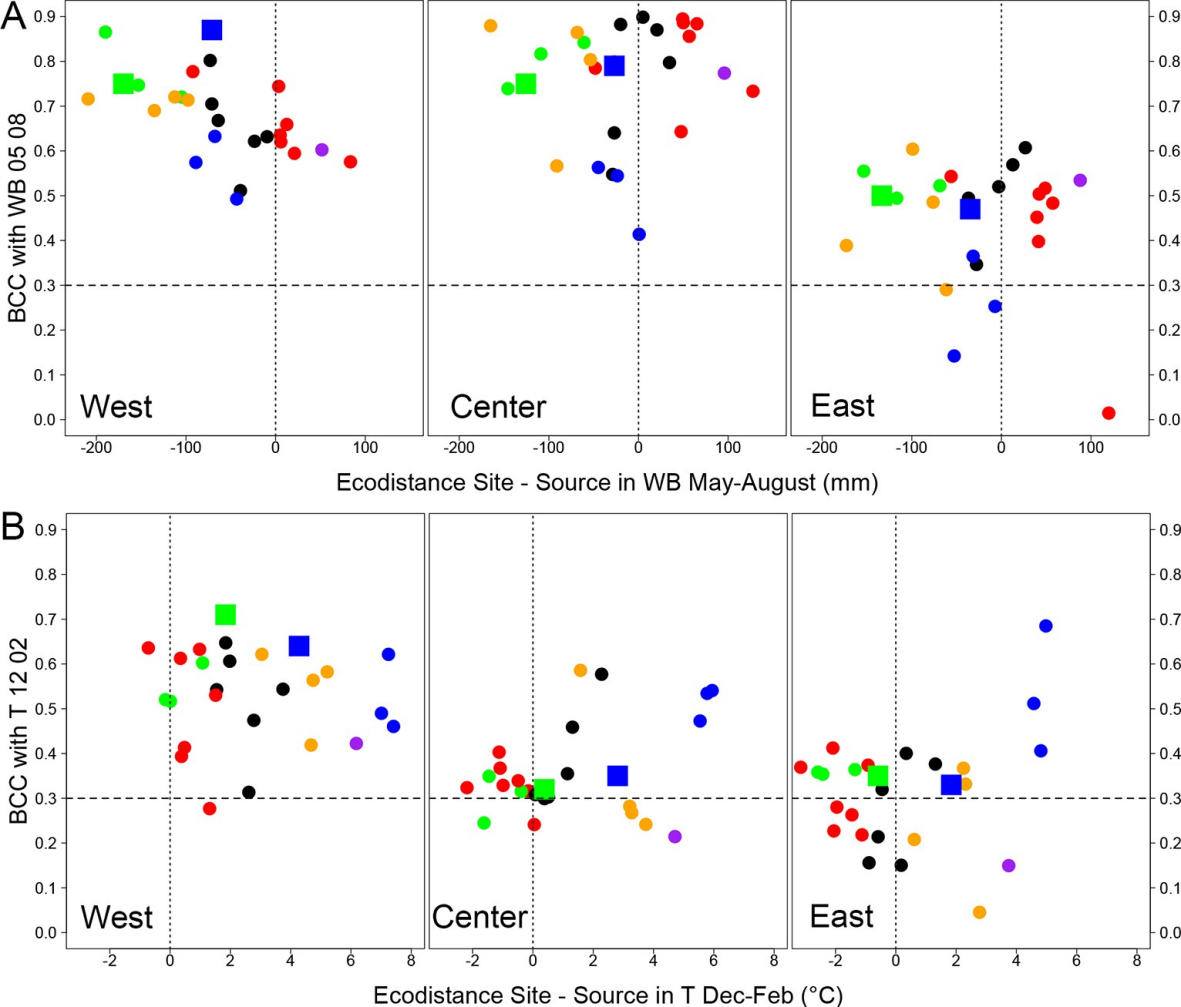
Bootstrapped correlation coefficients, by common garden and provenance. A. BCCs with cumulative water balance from May to August; a negative ecodistance indicates that the study site was drier than the site of origin. A high BCC indicates high sensitivity to water balance during the growing season. B. BCCs for mean temperature from the previous December to current-year February; a positive ecodistance means that the study site was warmer than the site of origin. Color codes represent the climatic groups (see Fig 1), and squares represent *Q*. *robur* provenances. The dashed horizontal line indicates the 5% threshold of significance for BCCs. The vertical dotted lines indicate an ecodistance of 0.

Under the driest climatic conditions (West), the correlation between growth and summer water balance (WB0506, WB0708) changed with ecodistance (Figs 6A and 7A). The linear regression between BCC and WB0508 had a significant negative slope for West (*p* = 0.003), and no significant slope for Center (*p* = 0.67) and East (*p* = 0.30). Thus, more negative ecodistances (i.e. conditions drier than those experienced by the source population) were associated with more positive BCCs (i.e. the provenances became more sensitive to water stress). Drought sensitivity was greatest for provenances originating from sites characterised by wet summers: Ireland and Great Britain (IRL_GB group) and Denmark/Germany/high-elevation French sites (DKDEFR) (Figs 1 and 7A).

All provenances were most sensitive to winter temperature (T1202) in the warmest common garden conditions, at West (Figs 6B and 7B). BCCs were lower at Center, and even lower at East, and were non-significant for about half the provenances. Pairwise comparisons of means performed by the Bonferroni method showed that BCCs based on T1202 were significantly higher at West than at Center (*p*<0.0001) and East (*p*<0.0001), with no significant difference between Center and East (*p* = 0.55). The relationships between growth and winter temperature were greater for warmer winter climates (Fig 4). The provenances from eastern European countries (Poland, Slovakia, Hungary) had high BCCs in the three common gardens.

BCCs analyses showed that temperature in July-August (T0708) was more negatively correlated with growth at West (Fig 6B). The mean BCC value at West (BCC = -0.37) was significantly different from those at Center (-0.16) and East (-0.16). However, no significant trend for the relationship between BCC and ecodistance was found within any of the sites. Thus, high summer temperatures were more limiting for radial growth for provenances planted at a dry site.

### Provenance effect within the original climatic groups

The geographic distribution of the 26 provenances was structured into six climatic groups (Fig 1). Within these groups, some provenances remained grouped together in the PCA on BCC, regardless of the common garden in which they were grown (S5 Fig), expressing an adaptation of oaks to the regional climate. This was the case for the eastern group, the IRL_GB group, provenances 250 Cochem (from southwest Germany) and 181 Horbylunde (Denmark), and provenances 5 Blois, 9 Saint-Sauvant and 10 Vouillé (from western France). By contrast, other climatic groups grouped together provenances with different responses to climate. For example, provenance 313 Bareilles (Pyrénées Mountains) had BCC values different from the three other provenances of the same climatic group (181 Horbylunde (Denmark), 225 Still (Vosges Mountains) and 250 Cochem (southwest Germany)). Thus, similarity of climate at the site of origin did not necessarily result in similar growth responses to climate at the same common garden site.

Similarly, geographic proximity of sites of origin did not necessarily imply similar responses and *vice versa*. For example, provenances 309 Bride (northeastern France) and 311 Prémery (central France) had similar responses to climate despite being obtained from sites 300 km apart. Conversely, provenances 29 Traconne (north central France) and 207 Fontainebleau (central France) were obtained from sites of origin only 75 km apart, but they presented similar BCCs at East, different BCCs at Center and very different BCCs at West. Finally, the growth response to climate was not always homogeneous within a climatic or geographic group, so the findings for the selected provenances cannot be extrapolated to entire regions.

### Species effect

This study focused primarily on *Quercus petraea* provenances, because this species was the most prevalent in the experimental tests. However, two provenances of *Quercus robur* were also sampled, making a brief comparison possible. One of the source populations was in the eastern climatic group and the other belonged to the IRL_GB group, so they were analyzed individually: (1) Provenance 47 was obtained from Fallersleben in Germany (65 km east of Hanover) and was included in the eastern climatic group. BCCs showed that this provenance behaved differently from *Q*. *petraea* from eastern countries and that its behavior more closely resembled that of *Q*. *petraea* from other climatic groups (Fig 7A). In particular, the negative effect of winter temperature on growth decreased from west to east for this provenance, but not for the other eastern provenances (Fig 7B). (2) Provenance 129 was from Drummond Castle (Scotland) and was compared with *Q*. *petraea* provenances from the IRL_GB climatic group. Very few differences were detected, and this *Q*. *robur* provenance had comparable BCCs to those of the other three British provenances.

Provenance 249 Bolu from Turkey was initially included as a *Q*. *petraea* provenance in the eastern climatic group (S2 Fig). In France, the provenances of this group were shifted to a warmer (+3 to +4.5°C in summer) and slightly drier climate, except for provenance 249, which experienced a WB 50 to 100 mm higher in May-August than at its site of origin (Fig 2). The correlations between growth and climate for provenance 249 Bolu were different from those of the other eastern provenances: (1) eastern provenances were generally less sensitive to drought, with BCCs lower than those of all other provenances, including 249 Bolu (Fig 7A); (2) provenance 249 Bolu displayed a stronger negative effect of water balance in September at the central common garden (Center), whereas this correlation was weaker for the other Eastern provenances (Fig 6A). The central common garden was more waterlogged than the others, and the Turkish provenance was less well adapted to these conditions; (3) provenance 249 Bolu was the only eastern provenance to display a significant positive effect of temperature in March-April on growth in the westernmost common garden (Fig 6A).

## Discussion

This study was based on three common gardens of *Quercus petraea* and *Q*. *robur* established in the northern half of France at the start of the 1990s. The source populations came from sites hundreds or thousands of kilometers away (even up to 2600 km for the provenance 249 Bolu). This design allowed to study the effects of two main factors on the relationship between climate and radial growth for each provenance: implantation site and displacement (ecodistance). The sampled oaks were 25 years old, and their growth responses to climate were therefore probably closer to those of mature oaks than to those of saplings [58] or very young trees [59]. With a view to developing recommendations for future reforestation, we also tried to group provenances according to their sites of origin in six homogeneous climatic areas in Europe or their dendroclimatic behavior. This clustering proved difficult, as plantation site appeared to have a stronger effect on growth response to climate than provenance.

### Effects of plantation site conditions

Plantation site strongly affected response to climate in three ways: (1) the most strongly correlated climate variables differed between sites (temperature or climatic water balance), (2) the strength of correlation (e.g., West vs. East), and (3) the most important months in the year (e.g., West vs. Center) differed between sites.

We found that the most important climate variables for radial annual growth were winter temperature and climatic water balance during the growing season (Fig 6). Water supply during the growing season has frequently been identified as an important factor in dendroecological studies, particularly for *Quercus petraea* [26, 29, 60]. Winter temperature was also positively correlated with growth in *Q*. *petraea* [27, 60, 61] and *Q*. *robur* [60, 61]. The processes responsible of such a correlation still need to be more investigated but some of these studies found that frosts reduce radial growth during the following months [60, 61] or change earlywood vessels area [62]. The large vessels of initial wood of Oaks are sensitive to winter (freezing of the xylem between 0 and -2°C). By reducing the percentage of embolized vessels, the mild winter temperatures allow maintaining the integrity of the driving system and therefore better growing conditions.

The correlation between climate and radial growth differed considerably between common gardens. Bootstrapped correlations for many provenances reached very high values between 0.5 and 0.9 for WB at West and Center (Fig 7A). These high correlation coefficients indicate that climatic variables accounted for 25% to 80% of the variance in radial growth during the 15-year study period. The percentage in natural stands studied over longer periods is frequently less than 20–25% [26, 28]. Stand competition is much weaker in common gardens than in productive forests, in which many other factors, in addition to climate, affect radial growth. For example, the correlations between growth and climate were not very different in natural forests on soils with highly contrasting water supply characteristics in Luxembourg [28]. Conversely, the homogeneous environmental conditions within each of our three common gardens highlighted differences in BCCs between common gardens. The mean BCC with water balance was much lower at East, where soil conditions were less limiting for water supply. Consistent with this finding, Friedrichs et al. [63] demonstrated that drier site conditions led to higher drought sensitivity in *Q*. *petraea* in central west Germany. In a previous study, it was possible to quantify the relative influences of the climate and soil of common gardens, and their interaction, on height growth in a network of 29 common gardens with contrasting soil properties [64]. In this large experiment, the provenances from colder sites of origin and higher elevations were more affected by climatic constraints, whereas soil and climate conditions were equally important for provenances from warmer sites of origin. Soil (Figs 6 and 7A) had a strong effect on the sensitivity of growth to climate, and the provenance effect was not strongly structured.

Ecodistance accounted poorly for the BCCs of most provenances (Fig 7). The common gardens may have been located too far north in France, and having some common gardens in the south might have enhanced the differences in climatic sensitivity between provenances. Nevertheless, ecodistance-based analyses revealed particular patterns of behavior in some groups of provenances: eastern provenances were highly sensitive to winter temperature in the three common gardens because they had been moved to much warmer sites, and IRL_GB and DKDEFR climatic group provenances were highly sensitive to climatic dryness at West because these provenances had been moved to sites much drier than their sites of origin. Similarly, Trouvé et al. [29] showed that sessile oaks at xeric sites recovered more rapidly after a dry year than trees at mesic and wet sites. This implies that trees growing at wetter sites are more vulnerable to drought events than those growing at drier sites. When acorns from trees acclimated to wetter conditions (e.g. the IRL_GB and DKDEFR groups in Figs 5 and 7A) were moved to drier common garden conditions (West), the resulting plants retained their higher sensitivity to drought than other provenances.

Ecodistance is generally based on geographic or climatic parameters and the response function at the population level often follows a “bell-shaped curve” that can be modelled with a quadratic function [11, 17, 65]. The response function is the expression of the plasticity of the species, allowing it to adapt to the environment at the planting site [22]. This pattern implies that the range of the species displays either no genetic structuration or that there is a uniform genetic gradient. In our case, genetic structuration was complex over the natural range [12] and a climatic ecodistance could not adequately represent the genetic variability and its associated response function to climate. The choice of provenances for the assisted migration of sessile oak will not, therefore, be driven by geographic or climatic parameters, provided that we remain within the limits of western continental European.

The climatic variables most strongly correlated with growth differed between common gardens (Fig 6A), because the important period of the year for oak functioning depends on soil characteristics. In particular, at Center, the WB of the preceding fall was positively correlated with growth, whereas this was not the case at the other two common gardens. There is no transpiration in winter due to the lack of foliage, so these variables may principally express the supply of water to the ground and its storage deep in the soil. A similar result was obtained for total precipitations in previous October-November, in place of WB1011, confirming that precipitation is the real factor expressed by water balance outside the growing season. This positive effect of soil water storage on oak growth during the following year has also been reported in the stagnosols of northeastern France [66]. This delayed effect of autumn water supply is stronger on earlywood formation than on latewood, which is more correlated with May-June precipitations [26]. Forest soils with a temporary water table occupy an area of about two million hectares in France. They have two disadvantages for tree growth: excess water in fall and winter due to poor drainage, and a shortage of water in summer due to the lack of a water table and shallow root systems [67]. Climate change may accentuate these problems, through decreasing levels of rainfall in summer and increasing levels of rainfall in winter [67] (Fig 2). In summary, the soil water cycle at Center was reflected in the BCC: positive correlation with the climatic water balance of the preceding fall (water stored in the soil for the following growing season), positive correlation with water balance during the growing season (stored and newly arriving water consumed for growth), negative correlation with water balance in September (excess water when the summer is too rainy).

A study of the effect of fertilizer on the radial growth of *Quercus petraea* in the vicinity of our westernmost common garden (West) showed that the soil water deficit in June to August was the factor most strongly affecting growth [68]. By contrast, Chakraborty et al. [64] found a predominant effect of climate, rather than soil, on the total height of 10-year-old *Picea abies* trees, over a much larger climate gradient. Taeger et al. [69] also showed that overall growth rates for *Pinus silvestris* in two provenance tests on similar soils in Germany were lower at the southern site, at which climatic conditions were drier. The differential response of provenances in a network of common gardens therefore represents a balance between the sensitivity of soil to drought and the climatic gradient covered.

### Provenances are unique

Several studies using different approaches and variables have reported considerable differences between individual populations, even neighboring stands, which may overlap with geographic effects [13, 55, 70, 71]. The conversion of geographic distances into ecodistances therefore made it easier to separate the genetic and environmental components of phenotypic variation between provenances [11, 23]. However, phenotypic plasticity is generally the predominant component of intraspecific variability, with a greater effect than local adaptation [72]. High plasticity for growth and survival traits confers an advantage on trees experiencing significant rapid changes to their environment at some stage in their lives. Conversely, plasticity buffers the differences in traits between provenances and makes it difficult to structure the differences in behavior in clustering analyses on provenances. Oak provenances must therefore be analyzed separately if they come from separate stands [13, 14]. The results of our work fully confirm these recommendations, although geographic groupings were possible for some climatic responses (e.g., British provenances, Eastern European provenances).

In the present study, the growth was found to be higher when the distance between the provenances and the common garden was lower. Similarly, in some early provenance studies (reviewed in [73]), populations growing close to the site of the provenance test were found to be more vigorous than other populations. Local provenances were therefore used as a reference for standardization of the trait studied to ensure comparability with other sites at which fertility was different. However, the superiority of local provenances has also been called into question in other studies (e.g., [74, 75]) and the concept of an “adaptation lag” was put forward in 1990 [76, 77]. Genetic selection and phenotype plasticity should be considered to function together. In a selection gradient, a population with a genotype not ideal for its environment may make use of its plasticity to deal with actual environmental conditions. When such populations are grown in a provenance test, the optimum may be slightly different, and the ranking of provenances may be modified.

Our sample also provided an overview of the effect of species on climate-growth relationships, because it included two provenances of pedunculate oak. Provenance 47 Fallersleben (central Germany) was much more vigorous than provenance 129 Drummond Castle (Scotland) (Fig 3). The growth of provenance 47 Fallersleben was more strongly correlated with summer water supply than that of *Q*. *petraea* from Eastern Europe and that of provenance 250 Cochem (south-west Germany). These findings are consistent with the differences between these two species generally reported in ecological and ecophysiological studies: *Q*. *robur* is more suitable for wet sites [24, 42, 60, 78], due to its lower water use efficiency (13% lower in [79]) in particular. This characteristic was largely responsible for the extensive dieback of *Q*. *robur* observed after the extreme drought in 1976 [80] and subsequent droughts in France [81]. However, no such differences were observed for the *Q*. *robur* provenance from Scotland because its climatic sensitivity was very similar to that of British *Q*. *petraea* provenances (Fig 7A), although it grew much more slowly than these provenances in the three common gardens.

In addition to specific dendroclimatic results, the Bolu provenance was known to display early bud break (in March), several weeks ahead of most of the other provenances (Ducousso, unpublished data). This source population was selected in the late 1980s as a *Quercus petraea* stand, and was planted at the common gardens. The morphology of the saplings was subsequently found to resemble that of what is now considered to be *Quercus petraea subsp*. *polycarpa* (Schur). Our findings confirm the particular pattern of this provenance, consistent with some of the ecological features specific to this subspecies [82].

### Which oak provenances for the 22^nd^ century?

**Fig 8:**
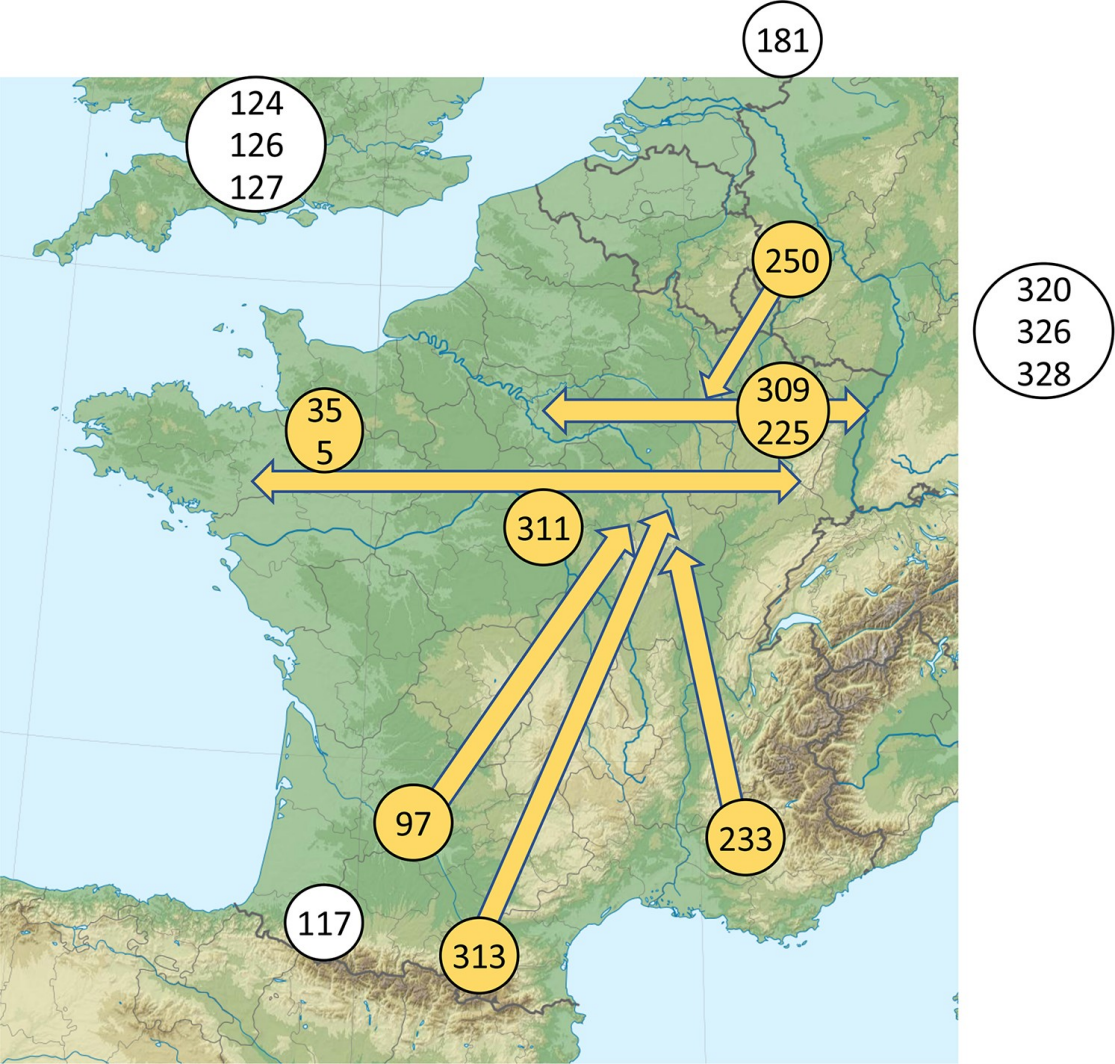
Map of western Europe with the sites of origin of some of the provenances studied and the main possible assisted migrations based on dendroclimatic results and growth rates. The orange arrows indicate the geographical trends but they require modulation according to the local environmental conditions. White circles indicate provenances potentially less well adapted to the predicted future climate of Western Continental Europe. (Licence for the map: http://artlibre.org/licence/lal/en/).

We show here that the response of oak to climate is highly dependent on soil characteristics. Moreover, the structuring of this response is weakly related to geography and climate at the European scale. This implies that recommendations should be carefully limited to the range of situations explored by the experiment: in this case mostly the northern half of France and the provenances studied. For future forest management in northern France, the most useful sessile oak provenances would display high growth rates and tolerances to various types of climatic stress, pathogens and biotic factors. Here, we aimed at characterize a subsample of sessile oak provenances from a large selection of provenances obtained from the main part of the natural range. Some of the French provenances were already considered to be “grands crus” (i.e., vintage provenances with good vigor and wood quality due to a high genetic potential and long-term silvicultural good practice). Such high quality had also already been reported for the trees of the Forest of Dean in England, represented here by provenance “127 Blakeney”. Such provenances proved their worth in the context of a previously stable climate. In the future, the climate sensitivity will need to be added to the parameters guiding forest managers in their selection of provenances for the next generation of oaks (Fig 8). Hereafter, we suggest the fate of the studied provenances according to (1) the climate sensitivity they showed in the period 1996–2010 and also according to (2) what is known today on possible future evolution of climate (Fig 2). The main climatic features taken in consideration were induced by future summer temperature that may change the climatic water balance during growing season by circa 0 to 50 mm (RCP 4.5 or RCP8.5 WRF), or circa -150 to -200 mm (RCP 8.5 Aladin) by 2100. The study also showed that winter temperature was linked to growth and the climate models and scenarios predict winter warming by 1 to 3.5°C by 2100. By synthesizing the past reactions of the provenances and the future evolution of the climate, in interaction with the soil, we thus seek to identify which provenances would be better adapted in Western Europe to be able to provide forest cover and wood in the 22nd century.

The DKDEFR climatic group consists of the four provenances for which the shift in climate corresponded to predicted future climatic scenarios: a drier growing season and a warmer winter (Fig 2). Provenance “181 Horbylunde” from Denmark should be avoided in future plantations, because it was not very vigorous and was highly sensitive to drought at West and Center. Provenance “225 Still” from the Vosges Mountains (north-east France) displayed similar features to “181 Horbylunde” at West, but grew well, with low sensitivity to drought, at the other two common gardens. Its use for reforestation could therefore still be envisaged, but not in the west of France. Provenances “250 Cochem” (south-west Germany) and “313 Bareilles” (Pyrénées Mountains, high elevation) grew well but were highly sensitive to drought at West and Center, displaying a lower sensitivity at East. They may potentially be compatible with the future climate in the environments of eastern France.

With respect to a shift towards warmer winters and a limited drier growing season (yellow gradient on Fig 2), the provenances from Eastern Europe “320 Kozienice”, “326 Obora” and “328 Nagybotany” were highly sensitive to winter temperature in the three common gardens. They were also sensitive to summer drought at West and Center. They displayed average growth at East and grew more slowly at the common gardens farther to the west. These provenances would not be well adapted for Western Europe in the future.

With respect to the summer climatic water balance in the absence of large changes in winter temperature (green gradient on Fig 2), the provenances from Britain were moved to much drier sites and were highly sensitive to summer drought at all three common gardens. Nevertheless, “127 Blakeney” (“grand cru”) and, to a lesser extent, “128 Coolgreany” (from Ireland) grew strongly for the first 10 years. Given their high sensitivity to water supply, it would be unwise to transfer them to Continental Europe in the future.

The other provenances were from France and their transplantation to the three common gardens studied represented only a slight shift in climate. Some displayed dynamic growth, but they also displayed various degrees of sensitivity to drought. They could still be used for forestry, provided that they survive future severe droughts with their high growth potential. Provenances “5 Blois” and “35 Bellême” were among the fastest growing provenances and are both considered to be “grand cru”. They were moderately sensitive to drought and insensitive to winter temperature in the three common gardens. They would be less well adapted to waterlogged soils than other provenances, because they were more sensitive to water stress at Center. The provenance “311 Prémery” (central France) is also a “grand cru” with good growth, moderate sensitivity to drought and moderate sensitivity to winter temperature at the three common gardens. The waterlogged soil seemed to be less stressful for “311 Prémery” than for “5 Blois” or “35 Bellême”, and would be suitable for use in many environments. Provenance “309 Bride” grew strongly at East, which is located close to its site of origin in northeast France. It also grew well at Center, but much less well at West. It was moderately sensitive to drought at Center and East and would therefore be more appropriate for use in the east of France rather than in the west, like “225 Still”.

Southern provenances are interesting because they come from places where the climate resembles that predicted for the future in the north. Our sample included three provenances from southern populations. Provenance “97 Grésigne” (from 50 km north of Toulouse) was highly sensitive to drought at West despite a mean climatic water balance similar to that at its site of origin, but less sensitive to drought at Center and East, where the water balance was less negative. It grew well, particularly at East, where the climate is colder. It could, therefore, be planted in northeast France. By contrast to most of the other provenances studied, provenance “233 Vachères” (south-east France) was moved to wetter climatic conditions. It had average or low levels of growth and was sensitive to drought at West and Center, but not East. Like “97 Grésigne”, it could potentially be used in north-eastern France, but its growth rate would be lower. Provenance “117 Adé” (foothills of the Pyrénées Mountains) was moved from a rainy climate to cooler and drier conditions. It was very sensitive to drought in all three common gardens, with average growth rates, and it was slightly sensitive to winter temperature. It would not be well adapted to conditions further north. Thus, the southern provenances came from dry (233, 97) or wet climates (117). All were sensitive to drought, particularly in the driest common garden, West. Both provenances originating from sites subject to climatic water stress have the potential for use further north. Their high sensitivity to climatic water balance suggests that they may be adapted to strong interannual variations of water supply rather than drought during years of water stress.

## Conclusions

Models based on climatic data suggest that the potential distribution range of European forest trees will shift northwards with climate change [18, 83]. Some tree populations may persist in their current locations for a while, depending on their sensitivity and ability to adapt to new environmental conditions, and for obvious socio-economic reasons relating to wood production. Forest managers have traditionally chosen provenances on the basis of their good growth, their morphology (architecture) and their low pathogen sensibility, but their climatic sensitivity has now become an important new issue. Forest managers have means of pragmatically mitigating these constraints by carefully selecting the provenances planted.

We found considerable variation between provenances in terms of climate-tree growth relationships, due to genotypic differentiation and strong interactions with local conditions in the zone of introduction (mostly climatic and soil characteristics). Such interactions conditioned the expression of adaptive processes, which were more pronounced at the most stressful sites [13, 59]. Great care is therefore required when choosing provenances and checking the ecological characteristics of the forested area. We identified some provenances better adapted to the predicted future climate at some sites, but transfers are subject to ecological limitations and caution is therefore required during assisted migration.

We measured the response to climate as an average over 15 years, but exacerbations may occur during years of strong climatic stress. The issue of resilience after strong climatic stress should therefore be considered, to build on the conclusions drawn here. Common gardens are valuable experimental tools, but most were implanted before climate change became a concern and they are often located within the usual range of the species. Consequently, extreme test locations are missing and, for oaks, sites at xeric limits would provide valuable information about the effects of the predicted future climate [84]. In particular, this study highlighted the important role of winter temperature: in mild winter climates oaks are more sensitive to cold. However, climate models predict significant winter warming, which could have consequences on future growth, in addition to the more usual predicted effects of a drier climate during the growing season. Overall, the provenances reacted differently and their responses were not well structured geographically. This implies that a large number of issues corresponding to many ecological situations will need to be studied.

## Supporting information

S1 Fig Diagram of the PCA, with axes 1 and 2, performed on the standardized climatic parameters T (temperature) and WB (water balance). The parameters are monthly for September (T09, WB09), and bi- or tri-monthly for the other months (e.g., WB0708 means July and August). The text in blue indicates corresponding climatic conditions.
(TIF)

S2 Fig Diagram of PCA, with axes 1 and 2, performed on the standardized climatic parameters T (temperature) and WB (water balance). The first dimension of the PCA (55.1%) was linked to the water balance during the growing season, and the second dimension (27.7%) expressed the mean temperature during the winter months. The code numbers of the provenances are shown in color according to climatic group. The text in blue indicates the climatic interpretation for the groups and the text in black indicates the meaning of the axes. Six principal groups, each consisting of four to seven provenances, were defined. The **TURKEY** group consisted of only one provenance due to the specific climate at an elevation of 1200 m: cold (7.2°C) and dry (650 mm). The **SW_France** group was the warmest, with a mean annual temperature (Tyear; mean 1970–2000 from WorldClim) of 11–12°C and a total annual precipitation (Pyear) of 650–1050 mm. The **NE_France** group was colder (9.5–11°C) and rather dry (600–900 mm). The **EAST** group, consisting of provenances from Germany, Poland, Slovakia and Hungary, was colder (8–9°C) and the driest (520–620 mm). The **DKDEFR** group, extending from the Pyrénées Mountains in southern France to Denmark, was also cold (7.6–11.2°C) but more rainy (700–1100 mm). Finally, the **IRL_GB** group of the British Islands had a mean temperature of 8.8–10°C and was rainy (850–1370 mm). The locations of the groups of source populations in Europe are plotted on Fig 1. The groups were used in the analyses of growth response to climate.
(TIF)

S3 Fig Time series of interannual variations of detrended radial growth (BAI) and detrended climatic water balance over summer (May, June, July and August) at the three common gardens for the provenance 233 Vachères from southern France. The Pearson coefficient and the BCC were 0.62 and 0,58, respectively, at the West common garden, 0.73 and 0.73 at Center (all statistically significant), 0.03 and 0.01 at East (not significant at the 5% threshold).
(TIF)

S4 Fig Sensitivity to drought according to vigor. Adjusted mean dimensions of provenances at 10 years of age, from the complete inventories for the three common gardens, taking into account the effects of stage. The numbers give the codes of the provenances included in this study. The codes for Center are shown in blue. The points are shown in color, according to the BCC with WB0508: gray for [0–0.3), light blue for [0.3–0.5), blue for [0.5–0.65), and black for [0.65–1].
(TIF)

S5 Fig Diagram of PCA, with axes 1 and 2, performed on the bootstrapped correlation coefficients (BCCs). The code numbers of the provenances are shown in color according to climatic group (see legend within the graphic). Codes: for 3_328, for example, the initial 3 is the code for the common garden (1 for West, 3 for Center, 4 for East) and 328 is the code for the provenance (Table 1).
(TIF)

S1 Table Number of sampled trees used in the analyses, by common garden and provenance, date of tree felling, age at this date, tree-ring series. (DOCX)

S2 Table Statistics of each master chronology by common garden. Rbar is the mean inter-series correlation and quantifies the strength of the signal common to all trees [56]. EPS is the Expressed Population Signal and quantifies the degree to which the chronology expressed the population chronology [56].
(DOCX)
