# Supplementary figures and images for: Which oak provenances for the 22nd century in Western Europe? Dendroclimatology in common gardens
Source: PLoS One. 2020 Jun 10;15(6):e0234583. doi: 10.1371/journal.pone.0234583 (PMC7286526; doi:10.1371/journal.pone.0234583)

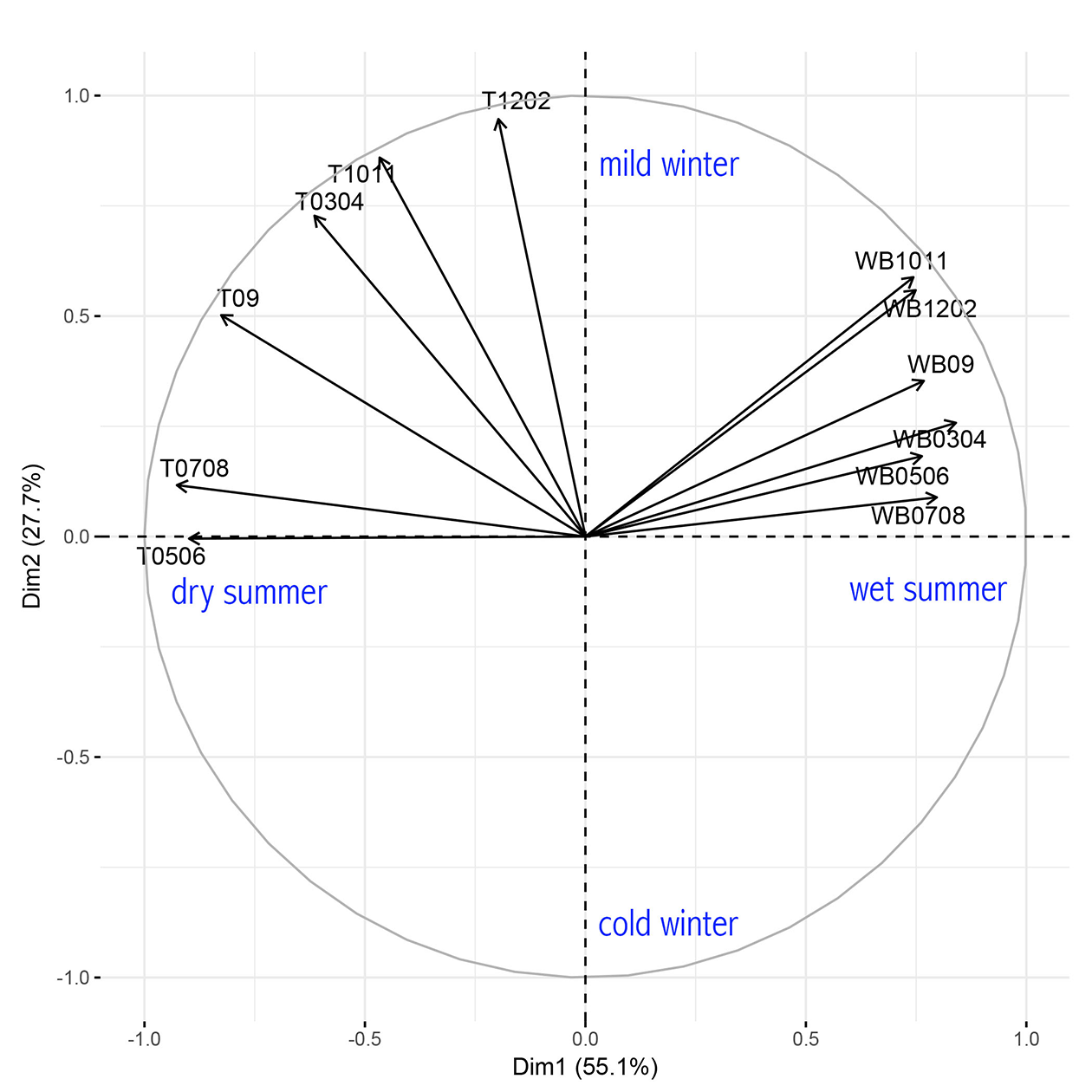

Supplement: S1 Fig — The parameters are monthly for September (T09, WB09), and bi- or tri-monthly for the other months (e.g., WB0708 means July and August). The text in blue indicates corresponding climatic conditions. (TIF) [file pone.0234583.s001.tif]

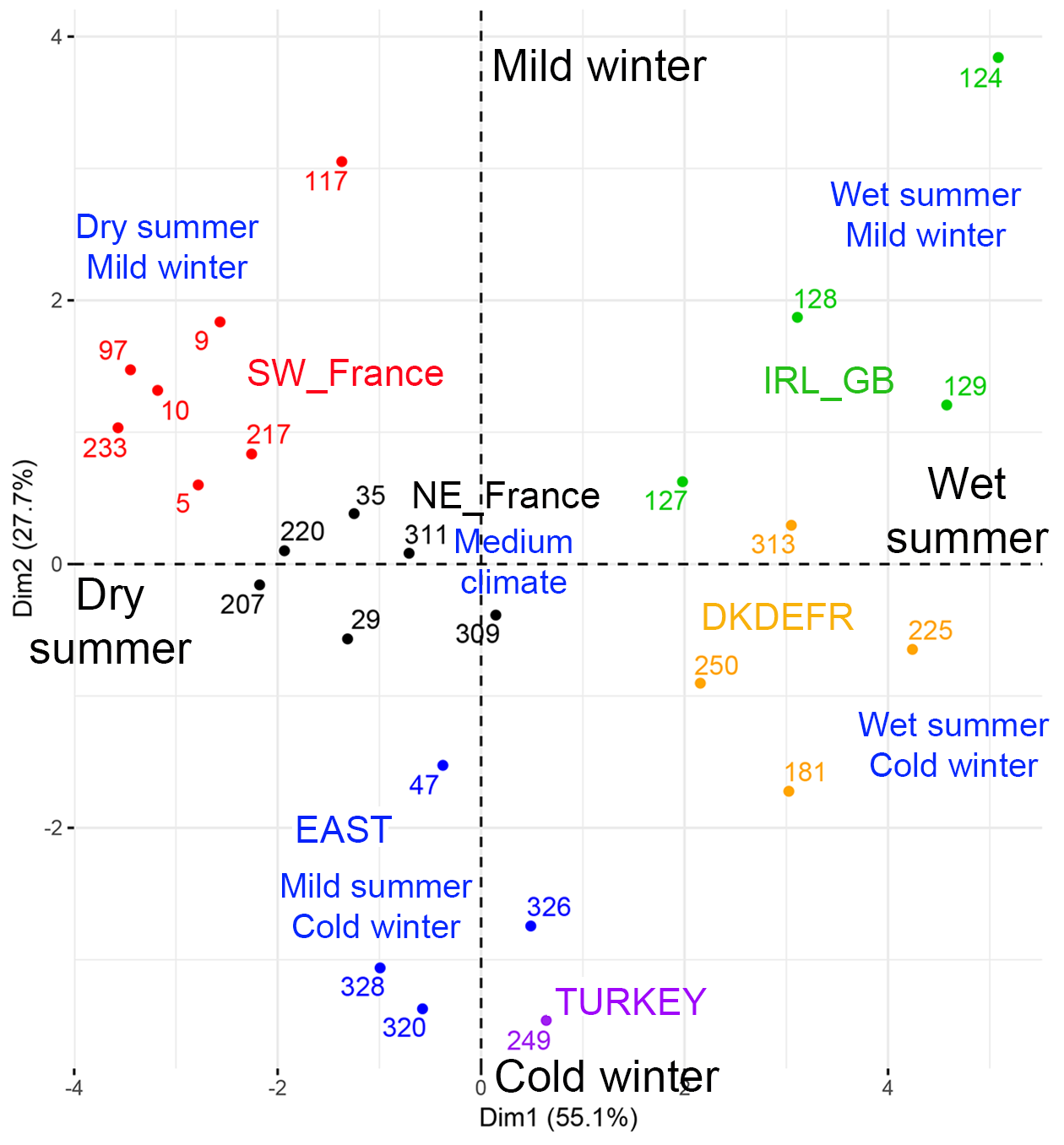

Supplement: S2 Fig — The first dimension of the PCA (55.1%) was linked to the water balance during the growing season, and the second dimension (27.7%) expressed the mean temperature during the winter months. The code numbers of the provenances are shown in color according to climatic group. The text in blue indicates the climatic interpretation for the groups and the text in black indicates the meaning of the axes. Six principal groups, each consisting of four to seven provenances, were defined. The TURKEY group consisted of only one provenance due to the specific climate at an elevation of 1200 m: cold (7.2°C) and dry (650 mm). The SW_France group was the warmest, with a mean annual temperature (Tyear; mean 1970–2000 from WorldClim) of 11–12°C and a total annual precipitation (Pyear) of 650–1050 mm. The NE_France group was colder (9.5–11°C) and rather dry (600–900 mm). The EAST group, consisting of provenances from Germany, Poland, Slovakia and Hungary, was colder (8–9°C) and the driest (520–620 mm). The DKDEFR group, extending from the Pyrénées Mountains in southern France to Denmark, was also cold (7.6–11.2°C) but more rainy (700–1100 mm). Finally, the IRL_GB group of the British Islands had a mean temperature of 8.8–10°C and was rainy (850–1370 mm). The locations of the groups of source populations in Europe are plotted on Fig 1. The groups were used in the analyses of growth response to climate. (TIF) [file pone.0234583.s002.tif]

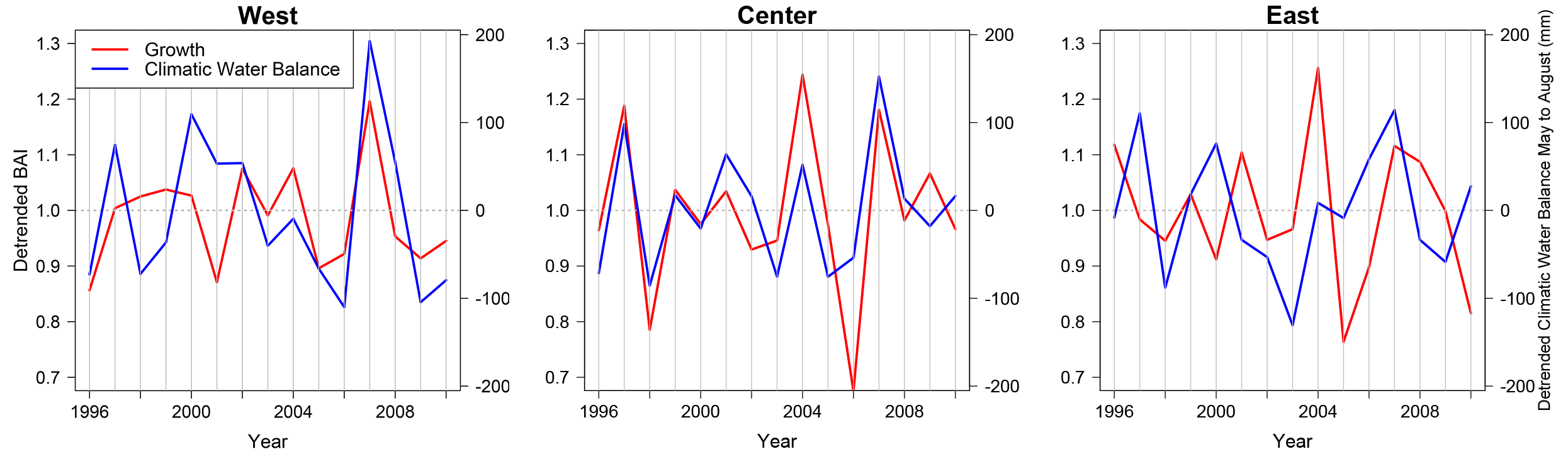

Supplement: S3 Fig — The Pearson coefficient and the BCC were 0.62 and 0,58, respectively, at the West common garden, 0.73 and 0.73 at Center (all statistically significant), 0.03 and 0.01 at East (not significant at the 5% threshold). (TIF) [file pone.0234583.s003.tif]

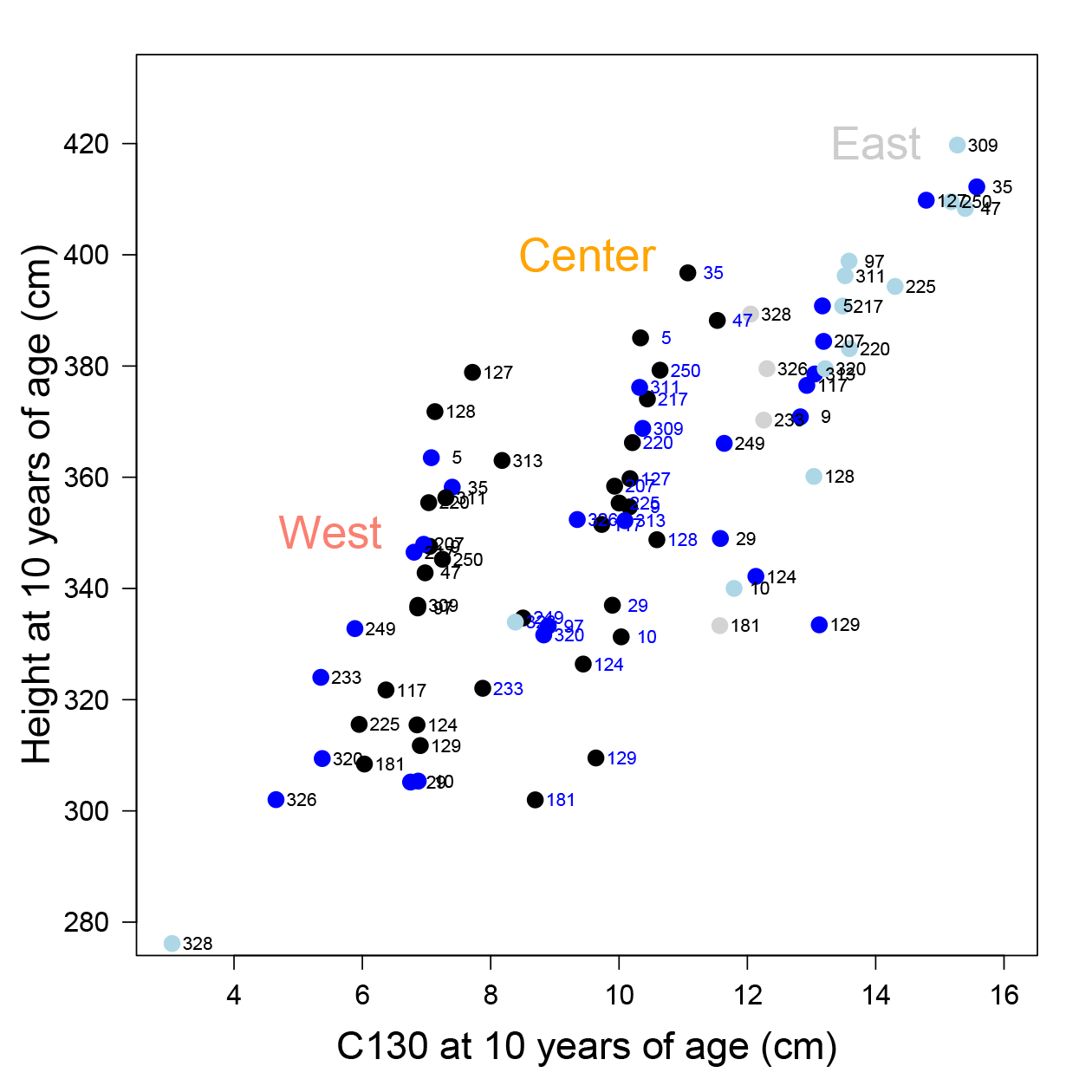

Supplement: S4 Fig — Adjusted mean dimensions of provenances at 10 years of age, from the complete inventories for the three common gardens, taking into account the effects of stage. The numbers give the codes of the provenances included in this study. The codes for Center are shown in blue. The points are shown in color, according to the BCC with WB0508: gray for [0–0.3), light blue for [0.3–0.5), blue for [0.5–0.65), and black for [0.65–1]. (TIF) [file pone.0234583.s004.tif]

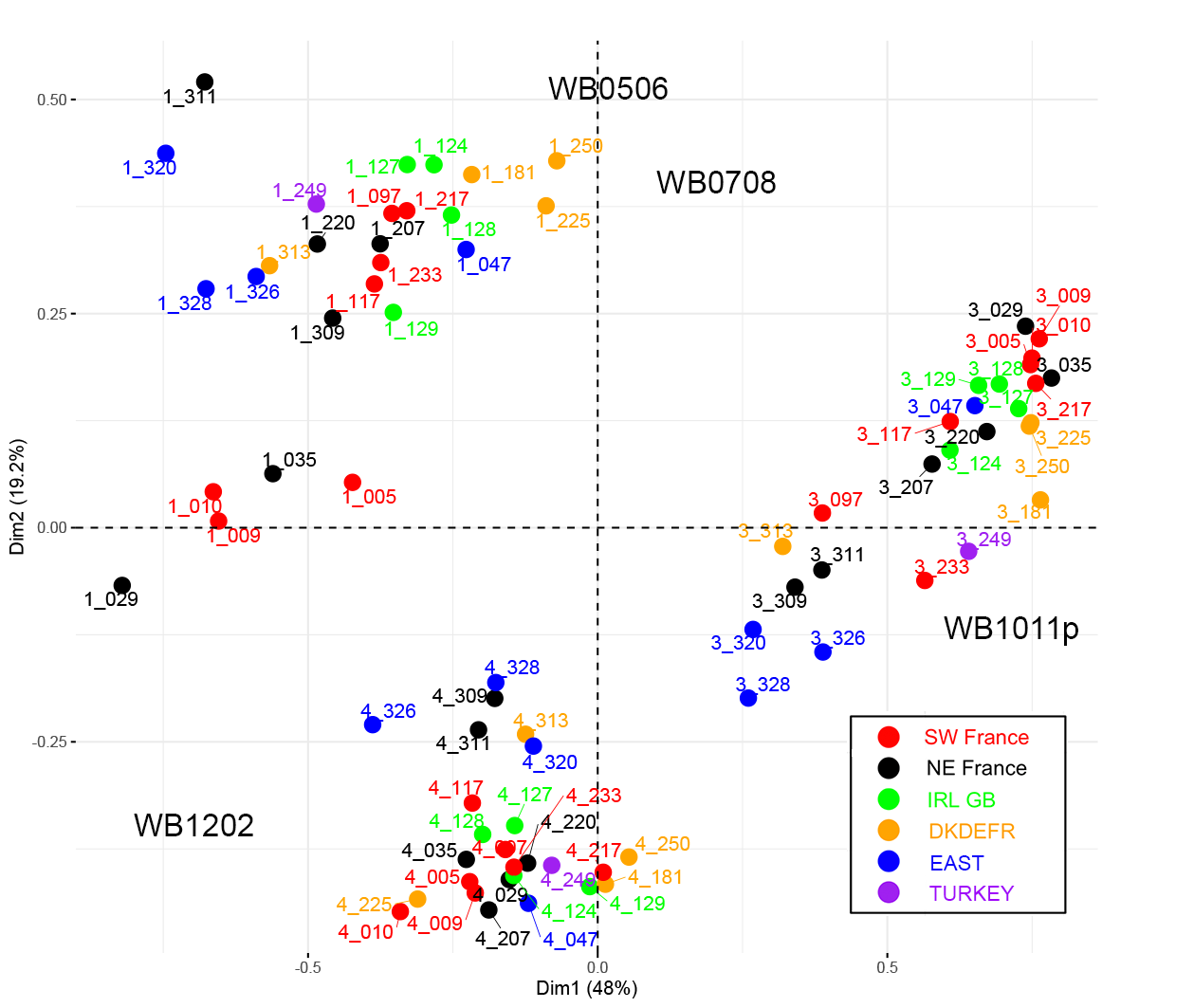

Supplement: S5 Fig — The code numbers of the provenances are shown in color according to climatic group (see legend within the graphic). Codes: for 3_328, for example, the initial 3 is the code for the common garden (1 for West, 3 for Center, 4 for East) and 328 is the code for the provenance (Table 1). (TIF) [file pone.0234583.s005.tif]
