# Supplementary material for: Which oak provenances for the 22nd century in Western Europe? Dendroclimatology in common gardens
Source: PLoS One. 2020 Jun 10;15(6):e0234583. doi: 10.1371/journal.pone.0234583 (PMC7286526; doi:10.1371/journal.pone.0234583)
Supplement: S1 Table — (DOCX) [file pone.0234583.s006.docx]

**S1_Table. Number of sampled trees used in the analyses,** by common garden and provenance, date of tree felling, age at this date, tree-ring series.

|  | | | Petite Charnie common garden (West) | | | | Vincence (Center) | | | | Sillegny (East) | | | |
| --- | --- | --- | --- | --- | --- | --- | --- | --- | --- | --- | --- | --- | --- | --- |
| Species | Code | Provenance | Stage 1 | Stage 2 | Stage 3 | Stage 4 | Stage 1 | Stage 2 | Stage 3 | Stage 4 | Stage 1 | Stage 2 | Stage 3 | Stage 4 |
| *Quercus petraea* | 5 | Blois | 41 |  |  |  | 25 |  |  |  | 35 |  |  |  |
|  | 9 | Saint-Sauvant | 42 |  |  |  | 20 |  |  |  | 22 |  |  |  |
|  | 10 | Vouillé | 29 |  |  |  | 18 |  |  |  | 26 |  |  |  |
|  | 29 | Traconne | 33 |  |  |  | 32 |  |  |  | 24 |  |  |  |
|  | 35 | Bellême | 47 |  |  |  | 18 |  |  |  | 31 |  |  |  |
|  | 97 | Grésigne |  | 24 |  |  |  | 16 |  |  |  | 31 |  |  |
|  | 117 | Adé |  | 24 |  |  |  | 35 |  |  |  | 38 |  |  |
|  | 124 | Killarney |  | 29 |  |  |  | 24 |  |  |  | 37 |  |  |
|  | 127 | Blakeney |  | 44 |  |  |  | 35 |  |  |  | 50 |  |  |
|  | 128 | Coolgreany |  | 33 |  |  |  | 34 |  |  |  | 33 |  |  |
|  | 181 | Horbylunde |  |  | 25 |  |  |  | 40 |  |  |  | 19 |  |
|  | 207 | Fontainebleau |  |  | 29 |  |  |  | 44 |  |  |  | 18 |  |
|  | 217 | Bercé |  |  | 23 |  |  |  | 47 |  |  |  | 20 |  |
|  | 220 | Dreuille |  |  | 26 |  |  |  | 37 |  |  |  | 21 |  |
|  | 225 | Still |  |  | 26 |  |  |  | 33 |  |  |  | 19 |  |
|  | 233 | Vachères |  |  | 26 |  |  |  | 29 |  |  |  | 17 |  |
|  | 249 | Bolu |  |  | 24 |  |  |  | 24 |  |  |  | 19 |  |
|  | 250 | Cochem |  |  | 29 |  |  |  | 44 |  |  |  | 20 |  |
|  | 309 | Bride |  |  |  | 28 |  |  |  | 44 |  |  |  | 48 |
|  | 311 | Prémery |  |  |  | 42 |  |  |  | 51 |  |  |  | 44 |
|  | 313 | Bareilles |  |  |  | 34 |  |  |  | 28 |  |  |  | 32 |
|  | 320 | Kozienice |  |  |  | 25 |  |  |  | 27 |  |  |  | 40 |
|  | 326 | Obora |  |  |  | 20 |  |  |  | 30 |  |  |  | 28 |
|  | 328 | Nagybotany |  |  |  | 14 |  |  |  | 23 |  |  |  | 24 |
| *Quercus robur* | *47* | *Fallersleben* |  | *28* |  |  |  | *42* |  |  |  | *49* |  |  |
|  | *129* | *Drummond Castle* |  | *42* |  |  |  | *41* |  |  |  | *47* |  |  |
| Total = 2420 trees | | **Trees** | **192** | **224** | **208** | **163** | **113** | **227** | **298** | **203** | **138** | **285** | **153** | **216** |
|  |  | Field sampling | Dec 2010 | Feb 2014 | Jan 2015 | Jan 2016 | Apr 2011 | Apr 2014 | Feb 2015 | Mar 2016 | Feb 2011 | Mar 2012 | Feb 2015 | Oct 2016 |
|  |  | Age | 24 | 26 | 25 | 23 | 24 | 26 | 25 | 23 | 24 | 24 | 25 | 24 |
|  |  | First tree-ring | 1987 | 1988 | 1990 | 1993 | 1987 | 1988 | 1990 | 1993 | 1987 | 1988 | 1990 | 1993 |
|  |  | Last tree-ring | 2010 | 2013 | 2014 | 2015 | 2010 | 2013 | 2014 | 2015 | 2010 | 2011 | 2014 | 2016 |
