# Supplementary material for: Which oak provenances for the 22nd century in Western Europe? Dendroclimatology in common gardens
Source: PLoS One. 2020 Jun 10;15(6):e0234583. doi: 10.1371/journal.pone.0234583 (PMC7286526; doi:10.1371/journal.pone.0234583)
Supplement: S2 Table — Rbar is the mean inter-series correlation and quantifies the strength of the signal common to all trees [56]. EPS is the Expressed Population Signal and quantifies the degree to which the chronology expressed the population chronology [56]. (DOCX) [file pone.0234583.s007.docx]

**S2_Table. Internal statistics of each master chronology** by common garden. Rbar is the mean inter-series correlation and quantifies the strength of the signal common to all trees [56]. EPS is the Expressed Population Signal and quantifies the degree to which the chronology expressed the population chronology [56].

|  | **Common garden** | **West** | | **Center** | | **East** | |
| --- | --- | --- | --- | --- | --- | --- | --- |
| Code | *Provenance* | rbar | EPS | rbar | EPS | Rbar | EPS |
| 5 | *Blois* | 0.33 | 0.95 | 0.63 | 0.97 | 0.56 | 0.98 |
| 9 | *Saint-Sauvant* | 0.36 | 0.96 | 0.32 | 0.90 | 0.59 | 0.97 |
| 10 | *Vouillé* | 0.44 | 0.95 | 0.68 | 0.97 | 0.59 | 0.97 |
| 29 | *Traconne* | 0.08 | 0.71 | 0.62 | 0.98 | 0.66 | 0.98 |
| 35 | *Bellême* | 0.40 | 0.97 | 0.74 | 0.98 | 0.60 | 0.98 |
| 47 | *Fallersleben* | 0.34 | 0.93 | 0.51 | 0.98 | 0.51 | 0.98 |
| 97 | *Grésigne* | 0.32 | 0.91 | 0.76 | 0.98 | 0.82 | 0.99 |
| 117 | *Adé* | 0.38 | 0.93 | 0.71 | 0.99 | 0.63 | 0.98 |
| 124 | *Killarney* | 0.25 | 0.90 | 0.73 | 0.98 | 0.75 | 0.99 |
| 127 | *Blakeney* | 0.37 | 0.96 | 0.71 | 0.99 | 0.54 | 0.98 |
| 128 | *Coolgreany* | 0.47 | 0.97 | 0.43 | 0.96 | 0.55 | 0.98 |
| 129 | *Drummond Castle* | 0.50 | 0.98 | 0.69 | 0.99 | 0.60 | 0.99 |
| 181 | *Horbylunde* | 0.29 | 0.91 | 0.35 | 0.95 | 0.39 | 0.92 |
| 207 | *Fontainebleau* | 0.46 | 0.96 | 0.62 | 0.98 | 0.29 | 0.87 |
| 217 | *Bercé* | 0.49 | 0.95 | 0.54 | 0.98 | 0.71 | 0.98 |
| 220 | *Dreuille* | 0.53 | 0.96 | 0.69 | 0.99 | 0.39 | 0.92 |
| 225 | *Still* | 0.44 | 0.95 | 0.50 | 0.97 | 0.51 | 0.95 |
| 233 | *Vachères* | 0.39 | 0.94 | 0.46 | 0.96 | 0.70 | 0.97 |
| 249 | *Bolu* | 0.22 | 0.86 | 0.45 | 0.95 | 0.33 | 0.9 |
| 250 | *Cochem* | 0.50 | 0.96 | 0.61 | 0.98 | 0.52 | 0.95 |
| 309 | *Bride* | 0.36 | 0.93 | 0.40 | 0.97 | 0.54 | 0.98 |
| 311 | *Prémery* | 0.43 | 0.97 | 0.37 | 0.97 | 0.48 | 0.97 |
| 313 | *Bareilles* | 0.30 | 0.93 | 0.46 | 0.96 | 0.32 | 0.93 |
| 320 | *Kozienice* | 0.29 | 0.91 | 0.44 | 0.95 | 0.43 | 0.97 |
| 326 | *Obora* | 0.18 | 0.79 | 0.44 | 0.96 | 0.43 | 0.95 |
| 328 | *Nagybotany* | 0.30 | 0.85 | 0.41 | 0.94 | 0.44 | 0.95 |
|  | Mean | 0.36 | 0.92 | 0.55 | 0.97 | 0.53 | 0.96 |
